# Risk of venous thromboembolism in patients with COVID‐19: A systematic review and meta‐analysis

**DOI:** 10.1002/rth2.12439

**Published:** 2020-10-13

**Authors:** Stephan Nopp, Florian Moik, Bernd Jilma, Ingrid Pabinger, Cihan Ay

**Affiliations:** ^1^ Clinical Division of Haematology and Haemostaseology Department of Medicine I Medical University of Vienna Vienna Austria; ^2^ Department of Clinical Pharmacology Medical University of Vienna Vienna Austria; ^3^ I.M. Sechenov First Moscow State Medical University (Sechenov University) Moscow Russia

**Keywords:** COVID‐19, prevalence, pulmonary embolism, severe acute respiratory syndrome coronavirus 2, venous thromboembolism

## Abstract

**Background:**

Venous thromboembolism (VTE) is frequently observed in patients with coronavirus disease 2019 (COVID‐19). However, reported VTE rates differ substantially.

**Objectives:**

We aimed at evaluating available data and estimating the prevalence of VTE in patients with COVID‐19.

**Methods:**

We conducted a systematic literature search (MEDLINE, EMBASE, World Health Organization COVID‐19 database) to identify studies reporting VTE rates in patients with COVID‐19. Studies with suspected high risk of bias were excluded from quantitative synthesis. Pooled outcome rates were obtained within a random effects meta‐analysis. Subgroup analyses were performed for different settings (intensive care unit [ICU] vs non‐ICU hospitalization and screening vs no screening) and the association of d‐dimer levels and VTE risk was explored.

**Results:**

Eighty‐six studies (33,970 patients) were identified and 66 (28,173 patients, mean age: 62.6 years, 60.1% men, 19.4% ICU patients) were included in quantitative analysis. The overall VTE prevalence estimate was 14.1% (95% confidence interval [CI], 11.6‐16.9), 40.3% (95% CI, 27.0‐54.3) with ultrasound screening and 9.5% (95% CI, 7.5‐11.7) without screening. Subgroup analysis revealed high heterogeneity, with a VTE prevalence of 7.9% (95% CI, 5.1‐11.2) in non‐ICU and 22.7% (95% CI, 18.1‐27.6) in ICU patients. Prevalence of pulmonary embolism (PE) in non‐ICU and ICU patients was 3.5% (95% CI, 2.2‐5.1) and 13.7% (95% CI, 10.0‐17.9). Patients developing VTE had higher d‐dimer levels (weighted mean difference, 3.26 µg/mL; 95% CI, 2.76‐3.77) than non‐VTE patients.

**Conclusion:**

VTE occurs in 22.7% of patients with COVID‐19 in the ICU, but VTE risk is also increased in non‐ICU hospitalized patients. Patients developing VTE had higher d‐dimer levels. Studies evaluating thromboprophylaxis strategies in patients with COVID‐19 are needed to improve prevention of VTE.

**Essentials**

- High rates of venous thromboembolism (VTE) have been reported in coronavirus disease 2019 (COVID‐19).
- We conducted a systematic review to estimate the VTE prevalence in patients with COVID‐19.
- A total of 22.7% of patients with COVID‐19 treated at the intensive care unit (ICU) suffer from VTE.
- Risk in non‐ICU hospitalized patients is substantial, and 8% develop VTE.

## 1 INTRODUCTION

The coronavirus disease 2019 (COVID‐19), caused by the severe acute respiratory syndrome coronavirus 2 (SARS‐CoV‐2) and formally declared a pandemic by the World Health Organization (WHO) in March 2020, is an infectious disease with a global impact on public health. It affects primarily the respiratory system; however, involvement of other organ systems may occur, especially with increasing severity of the disease. The high inflammatory burden associated with COVID‐19 and inflammation in the vascular system can also result in cardiovascular complications with a variety of clinical presentations.^1^, ^2^, ^3^ Early studies reported on coagulation abnormalities and coagulopathy with a rather prothrombotic phenotype in patients with COVID‐19. ^4^, ^5^

With the better understanding of COVID‐19 and its clinical course, venous thromboembolism (VTE), a disease entity covering pulmonary embolism (PE) and deep vein thrombosis (DVT), has been recognized as a particular complication of the disease. Initial studies have found alarmingly high rates of PE in patients with severe COVID‐19 treated at intensive care units (ICUs), reporting VTE incidences of up to 50%.^6^ In response to the clinical challenges and the absence of high‐quality evidence, expert groups and scientific societies have released guidance statements to address questions concerning diagnosis, prevention, and treatment of VTE in patients with COVID‐19, which suggest the broad application of thromboprophylaxis in patients with severe COVID‐19 in the absence of high bleeding risk.^7^, ^8^

In several studies of different design, size, and quality, rates of VTE in patients with COVID‐19 have been reported. However, a definitive and robust estimate of the VTE risk in patients with COVID‐19 is currently not available as of the high variability of reported rates. Therefore, the true underlying burden of VTE in patients with COVID‐19 is still not fully understood. In the light of the ever‐growing infection rates worldwide and the clinical challenges in patient management, understanding of the true frequency of VTE in COVID‐19 is important and may help to support clinical decision making.

We conducted a systematic review of the literature and meta‐analysis of available data to determine the prevalence of VTE in patients with COVID‐19. Our aim was to provide an overall estimate of VTE by aggregating reported rates and to thoroughly explore differences in the VTE prevalence according to study design and the health care setting, which may account for the high degree of heterogeneity in reported rates.

## 2 METHODS

### 2.1 Register and protocol

We conducted a systematic review of the literature and meta‐analysis of published data on the prevalence of VTE in patients with COVID‐19. The study protocol was prepared before the initiation of the literature research according to the Preferred Reporting Items for Systematic Review and Meta‐analysis (PRISMA) Protocols 2015 ^9^ and submitted to PROSPERO (international prospective register of systematic reviews) on June 11, 2020 (protocol ID: CRD42020191652). The study was conducted according to the PRISMA and the guidance for reporting meta‐analysis of observational studies in epidemiology.^10^, ^11^

### 2.2 Eligibility criteria

Full‐text articles, letters, brief reports, editorials, and correspondences published in 2019 or 2020 with available title and abstract in English were eligible for inclusion. Inclusion criteria comprised studies reporting on patients with objectively confirmed COVID‐19 in combination with reporting rates of VTE as outcome of the study (DVT and/or PE). Study designs eligible for inclusion were cohort studies (prospective and retrospective), cross‐sectional studies, and interventional studies with VTE reported as an outcome or adverse event. Study designs that did not allow prevalence estimates such as case reports and case series including autopsy studies were excluded.

### 2.3 Literature research

**Figure 1:**
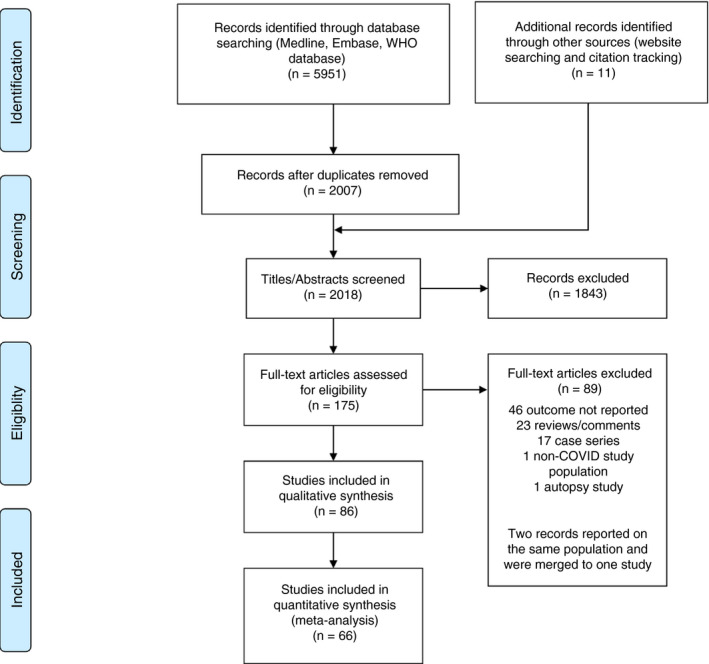
Preferred Reporting Items for Systematic Reviews and Meta‐Analyses (PRISMA) flow diagram for study selection

We systematically searched EMBASE, MEDLINE, and the WHO COVID‐19 research database with distinct predefined search algorithms to identify relevant publications. The exact search protocol is available in the Supplementary Methods. Search for additional studies not identified by the search criteria (eg, due to preprint status) was conducted by inquiring databases of preprint servers (medRxiv) and by manual research of relevant journals. Publications in preprint status were eligible only if they had undergone full peer review at the date of literature research. Duplicate search results were excluded before eligibility screening. Two researchers (SN, FM) screened title and abstract of the identified studies, and potentially eligible studies underwent full‐text evaluation. The inclusion of a study was based on the consensus of its suitability by the two researchers. Where consensus opinion could not be reached, a third reviewer was consulted to make the final decision (CA). All three literature researchers are medical doctors with a thorough research background in the field of thrombosis. The most recent literature research was conducted on August 26, 2020. Figure 1 displays the process of study identification following a PRISMA flow diagram.

### 2.4 Data extraction

Studies that fulfilled the predefined inclusion criteria and did not meet any exclusion criteria were subjected to data extraction. In the case of multiple studies reporting on the same patient cohort, results were merged and considered only once. Data extraction of predefined baseline and outcome variables was performed. These included methodological specifics of the studies (study design, health care setting), clinical information of the study population (demographics, comorbidities, disease severity, use of pharmacologic thromboprophylaxis, ultrasound screening, and d‐dimer levels), and outcome specifics (definition, type, and rate of VTE). The full list of extracted variables is provided in the Supplementary Methods. All data were independently extracted from eligible studies by two authors (SN, FM) to ensure data reliability, with inconsistencies resolved by discussion with a third author (CA).

### 2.5 Risk of bias evaluation

Methodology of identified studies was assessed independently by two researchers (FM, SN). Risk of bias of included studies was independently rated with a validated tool for assessing studies reporting prevalence data (Joanna Briggs Institute Critical Appraisal Checklist; Appendix S1). ^12^ This tool consists of nine categories each classifying the study as low risk of bias, high risk of bias, or unclear. Subsequently, an overall evaluation based on these categories was derived. Studies with suspected high risk of bias were excluded from the subsequent quantitative data synthesis. Potential publication bias was assessed graphically within a funnel plot, plotting the prevalence estimate of VTE against its standard error (Figure S1A and B).

### 2.6 Outcomes, definitions, and quantitative data synthesis

The primary outcome of the present meta‐analysis is VTE, defined as DVT (including catheter‐related thrombosis), PE, or the composite of both, as defined within the respective study. Thrombotic occlusions of mechanical components of extracorporeal devices such as dialysis machines or extracorporeal membrane oxygenation devices were not counted as outcome event. The prevalence estimate of the primary outcome is reported stratified by the use of systematic ultrasound screening for thrombosis in the respective studies.

Secondary outcomes included (i) the pooled prevalence of VTE (excluding studies reporting only isolated PE or isolated DVT rates), (ii) the pooled rate of PE, and (iii) the pooled rate of DVT. Outcomes of the secondary analyses were reported stratified for ICU patients and non‐ICU hospitalized patients at study baseline and by the performance of DVT screening. The ICU cohort comprised patients admitted to the ICU, or alternatively those who were defined as being critically ill, or in need of mechanical ventilation at baseline. Further, an exploratory analysis of differences between baseline levels of d‐dimer between patients experiencing VTE and those who did not was conducted.

Outcome definitions throughout the different studies were varying. Some studies reported pure incidence, while others reported prevalence, including patients who were admitted due to VTE and COVID‐19. In this systematic review, we have decided to aggregate the proportion of patients who have been diagnosed with VTE as reported in the included studies.

### 2.7 Statistical methods

All statistical analyses were performed with the commercially available package STATA 15.0 (Stata Corp, Houston, TX, USA). Summary statistics were aggregated from included studies. Pooled prevalence of outcome variables was estimated by aggregating study results within a random‐effects meta‐analysis utilizing the STATA package *metaprop*. ^13^ The Freeman‐Tukey double arcsine transformation was used to normalize variance, and 95% confidence intervals (CIs) were estimated by the score method. Heterogeneity of included studies is reported by I^2^ as a measure of between‐study variability beyond random variation. To explore differences in baseline d‐dimer between patients with VTE and patients without VTE, mean d‐dimer levels and corresponding standard deviation were calculated from reported median, interquartile range, and sample size according to Wan et al.^14^ Weighted mean differences (WMDs) in baseline d‐dimer levels were calculated within a pooled analysis weighted by corresponding sample sizes. Finally, differences in VTE risk according to sex and comorbidities was explored within a random‐effects meta‐analysis using the Mantel–Haenszel procedure.

## 3 RESULTS

### 3.1 Selection process and general study characteristics

We identified 2018 records upon literature research after the removal of duplicates. Titles and abstracts of these identified studies were screened for conformity with our predefined inclusion and exclusion criteria, and 175 records were subsequently included in the full‐text evaluation. From those, 86 studies were included in the qualitative data synthesis. Figure 1 displays the screening and selection process, and the reasons for excluding studies.

Pooled summary characteristics of the 86 eligible studies reporting on VTE in patients with COVID‐19 are displayed in Table 1. Regarding geographic regions, 57 studies were performed in Europe, 17 in North America, 8 in Asia, and 1 in Africa, and 3 studies included patients from multiple continents. Fifty‐eight cohort studies, 5 cross‐sectional studies, and 2 case‐control studies were carried out to identify the rate of VTE in the study populations, 15 cohort studies, and 2 case‐control studies reported VTE as a secondary outcome, and 4 studies reported VTE as an adverse event. VTE screening (ultrasound examination of deep veins of the upper and/or lower extremities) was performed in 19 studies, with 1 study conducting ultrasound screening in 28% of patients. ^15^ Twenty‐four studies were conducted specifically in ICU cohorts only, and 19 studies reported rates of VTE separately for ICU or critically ill patients, rendering 43 studies eligible for our ICU subgroup analysis.

**Table 1:** Characteristics of identified studies

	No. of studies	No. of patients
Study location		
Europe	57	11 709
North America	17	17 127
Asia	8	1962
Multinational and other	4	3172
Study design		
Randomized controlled trial	2	1296
Cohort study	75	28 536
Cross sectional study	5	502
Case‐control study	4	3636
Institutional setting		
Single center	64	20 729
Multicenter	22	13 241
Health care setting		
Ambulatory and hospitalized	9	4773
Hospitalized (± ICU)	53	27 155
ICU	24	2042
Reported outcomes		
Overall VTE	50	20 961
PE	61	22 618
DVT	54	20 773
VTE screening		
Yes	19	1440
No	59	27 106
Not reported	8	5424
Use of anticoagulation (either prophylactic or therapeutic)		
100% of patients	34	3312
>90% of patients	7	1762
<90% of patients	10	4681
Not reported	35	24 215

Abbreviations: DVT, deep vein thrombosis; ICU, intensive care unit; PE, pulmonary embolism; VTE, venous thromboembolism.

A comprehensive summary of each study including the respective study design, demographics, thromboprophylaxis strategy, and outcome rates is presented in Tables S1 and S2.

Pooled patient characteristics and comorbidity data are displayed in Table 2. The overall weighted mean age of patients was 62.6 years (SD, 3.8), and 60.1% were male. Weighted mean age of patients in ICU‐only studies was 62.6 years (SD, 2.9), and 71.3% were male.

**Table 2:** Patient characteristics

	No./Total (%) of patients	No./Total (%) of ICU/critical care patients
Mean age (±SD) in years	**62.6** (±3.8)	**62.6** (±2.9)
Sex		
Male	11 817/19 671 **(60.1)**	1632/2321 **(71.3)**
Female	7854/19 671 **(39.9)**	689/2321 **(29.7)**
Hypertension	6446/12 583 **(51.2)**	779/1509 **(51.6)**
Dyslipidemia	2993/8330 **(35.9)**	177/436 **(40.6)**
Diabetes mellitus type 2	4088/13 361 **(30.6)**	533/1748 **(30.5)**
Current or former smoker	985/7421 **(13.3)**	214/899 **(23.8)**
Cancer	805/7979 **(10.1)**	90/965 **(9.3)**
Active cancer	55/1509 **(3.6)**	20/587 **(3.4)**
Chronic kidney disease	1024/8101 **(12.6)**	136/1328 **(10.2)**
Coronary artery disease	1693/10 622 **(15.9)**	132/979 **(13.5)**
Congestive heart failure	865/9612 **(9.0)**	49/786 **(6.2)**
Chronic liver disease	85/3011 **(2.8)**	42/839 **(5.0)**
Chronic lung disease	1214/9728 **(12.5)**	162/1233 **(13.1)**
Prior VTE	321/7392 **(4.3)**	40/699 **(5.7)**
Cardiovascular disease	412/1198 **(34.4)**	249/706 **(35.2)**
Cerebrovascular disease	182/2282 **(8.0)**	42/411 **(10.2)**
Immune disease or immunosuppression	175/2456 **(7.1)**	49/629 **(7.8)**
Asthma	208/2120 **(9.9)**	58/480 **(12.1)**

Abbreviations: ICU, intensive care unit; VTE, venous thromboembolism.

### 3.2 Risk of bias

Risk of publication bias was evaluated separately for studies on non‐ICU hospitalized and ICU patients to enhance interpretability. Upon visual inspection of the funnel plots, no indication for publication bias was detected, with outliers in the distribution being explained by differences in ultrasound screening strategies (Figures S1A and B) Second, we conducted an exploration of potential time dependencies in VTE rates of published studies suggesting a decrease of VTE rates over time upon visual inspection and fitting a regression line of the VTE rate and the last patient inclusion date of each respective study (Figure S2).

Third, a methodological assessment of included studies was conducted to evaluate the risk of underlying bias regarding the reported rate of VTE. Importantly, this evaluation is not to be regarded as a general evaluation of quality and goodness of included studies but rather an evaluation of the generalizability of reported VTE rates.

In our quality assessment, low risk of bias was attributed to our identified studies in median in seven of nine categories (range, 3‐9, maximum: low risk of bias in all nine categories). The results of our structured methodological assessment of all 86 studies are presented in Table S3. In consensus among the three reviewers, 20 studies were excluded from quantitative synthesis upon a strong suspicion of bias in the structured assessment. Reasons for exclusion include selection bias (18 studies), reporting/information bias (1 study), and lack of background information on setting and outcomes (1 study). Therefore, the 66 remaining studies (including 43 studies reporting on ICU patients and 43 studies reporting on non‐ICU hospitalized patients) were included in quantitative data synthesis. ^6^, ^16^, ^17^, ^18^, ^19^, ^20^, ^21^, ^22^, ^23^, ^24^, ^25^, ^26^, ^27^, ^28^, ^29^, ^30^, ^31^, ^32^, ^33^, ^34^, ^35^, ^36^, ^37^, ^38^, ^39^, ^40^, ^41^, ^42^, ^43^, ^44^, ^45^, ^46^, ^47^, ^48^, ^49^, ^50^, ^51^, ^52^, ^53^, ^54^, ^55^, ^56^, ^57^, ^58^, ^59^, ^60^, ^61^, ^62^, ^63^, ^64^, ^65^, ^66^, ^67^, ^68^, ^69^, ^70^, ^71^, ^72^, ^73^, ^74^, ^75^, ^76^, ^77^, ^78^, ^79^, ^80^, ^81^

### 3.3 Prevalence of venous thromboembolism

**Figure 2:**
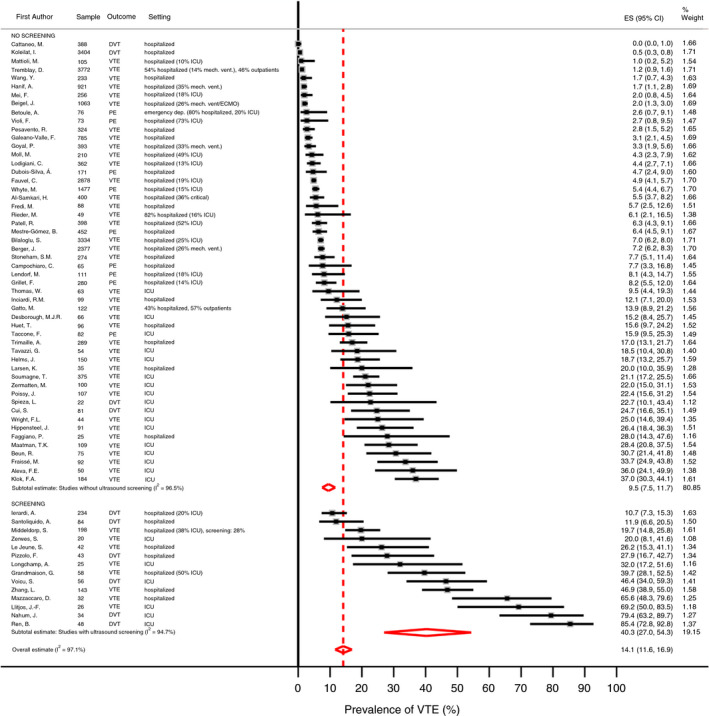
Prevalence of VTE in patients with COVID‐19. Prevalence of VTE is estimated based on 66 studies and stratified by the performance of ultrasound screening for VTE. The overall VTE prevalence was 14.1% (95% CI, 11.6‐16.9), 40.3% (95% CI, 27.0‐54.3) in those screened and 9.5% (95% CI, 7.5‐11.7) in those not screened. Red diamonds represent subtotal (screening studies vs nonscreening studies) and overall prevalence estimates and corresponding 95% CI of VTE outcomes. VTE comprises the specific outcome as reported by the respective study (PE and/or DVT). Details on each study are listed in Tables S1 and S2. CI, confidence interval; DVT, deep vein thrombosis; ECMO, extracorporeal membrane oxygenation; ES, estimates; mech. vent., mechanically ventilated; ICU, intensive care unit; PE, pulmonary embolism; VTE, venous thromboembolism

After excluding studies with a high risk of underlying bias, quantitative results from 66 studies were aggregated within a meta‐analysis, including 28 173 patients (1819 ambulatory, 20 886 non‐ICU hospitalized, 5468 ICU patients). In total, 1824 VTE events were reported. The pooled prevalence estimate of all reported VTE events (outcomes: VTE, DVT, or PE) was 14.1% (95% CI, 11.6‐16.9; I^2^, 97.1%). In the 52 studies (n = 27 130; 1492 VTE) in which no ultrasound screening was performed, the estimated rate of VTE was 9.5% (95% CI, 7.5‐11.7; I^2^, 96.5%). Conversely, in the 14 studies with ultrasound screening performed (n = 1 043; 332 VTE), the estimated prevalence of VTE was 40.3% (95% CI, 27.0‐54.3; I^2^, 94.7%). Figure 2 shows a forest plot of VTE rates, together with information on health care setting, the performance of screening, and outcome definition of respective studies.

### 3.4 Prevalence of VTE in hospitalized and ICU patients

The rates of VTE within our primary analysis strongly differed among studies, depending on the specifics of the study setting, design, and outcome definition. Therefore, to further explore heterogeneity of the reported VTE rates, we conducted detailed subgroup analyses based on the health care setting (non‐ICU hospitalized vs ICU patients), and the performance of DVT screening (screening vs no screening). In addition, within these subgroup analyses, we have separately estimated rates of VTE, PE, and DVT.

In 43 studies reporting on ICU cohorts including 5468 patients, the rate of VTE, PE, or DVT was available. The estimated prevalence of VTE, PE, and DVT was 22.7% (95% CI, 18.1‐27.6; I^2^, 87.3%), 13.7% (95% CI, 10.0‐17.9; I^2^, 87.6%), and 18.7% (95% CI, 12.6‐25.6; I^2^, 94.6%). Rates of VTE and DVT in studies with screening strategies in the ICU cohorts (9 studies, n = 359) were 45.6% (95% CI, 30.6‐61.1; I^2^, 73.4%) and 48.5% (95% CI, 31.0‐66.2; I^2^, 91.0%), and in those without screening 18.7% (95% CI, 14.9‐22.9; I^2^, 83.1%) and 8.9% (95% CI, 5.8‐12.4; I^2^, 86.2%).

In the meta‐analysis of studies reporting on non‐ICU hospitalized patients at baseline, including 20 886 patients from 43 studies, prevalence estimates of VTE, PE, and DVT were 7.9% (95% CI, 5.1‐11.2; I^2^, 94.6%), 3.5% (95% CI, 2.2‐5.1; I^2^, 88.9%), and 4.1% (95% CI, 2.3‐6.4; I^2^, 94.6%), respectively. In studies with ultrasound screening performed (8 studies, n = 684), rates of VTE and DVT were 23.0% (95% CI, 3.2‐52.5; I^2^, 96.5%) and 12.7% (95% CI, 3.7‐25.5; I^2^, 94.1%), respectively, compared to 5.5% (95% CI, 3.6‐7.9; I^2^, 91.0%) and 1.4% (95% CI, 0.7‐2.3; I^2^, 85.0%) in studies without screening. The results of these subgroup analyses are summarized in Table 3, and corresponding forest plots are available in Figures S3A and B.

**Table 3:** Prevalence of venous thromboembolism, pulmonary embolism and deep vein thrombosis in ICU and non‐ICU hospitalized patients with COVID‐19

Outcome	Studies	Number of patients	Number of outcomes	Estimate of prevalence, % (95%CI)	Heterogeneity (I^2^)
ICU patients only					
VTE (studies reporting both outcomes)	25	2966	617	22.7 (18.1‐27.6)	87.3
No Screening	20	2791	535	18.7 (14.9‐22.9)	83.1
Screening^a^	5	175	82	45.6 (30.6‐61.1)	73.4
PE (±DVT)^b^	27	3085	410	13.7 (10.0‐17.9)	87.6
DVT (±PE)	28	3001	423	18.7 (12.6‐25.6)	94.6
No Screening	19	2642	251	8.9 (5.8‐12.4)	86.2
Screening^a^	9	359	172	48.5 (31.0‐66.2)	91.0
Non‐ICU hospitalized patients^c^					
VTE (studies reporting both outcomes)	23	7390	411	7.9 (5.1‐11.2)	94.6
No Screening	19	7053	321	5.5 (3.6‐7.9)	91.0
Screening^a^	4	337	90	23.0 (3.2‐52.5)	96.5
PE (±DVT)^b^	23	8698	263	3.5 (2.2‐5.1)	88.9
DVT (±PE)	22	10 519	256	4.1 (2.3‐6.4)	94.6
No Screening	14	9835	144	1.4 (0.7‐2.3)	85.0
Screening^a^	8	684	112	12.7 (3.7‐25.5)	94.1

Note

The meta‐analysis of VTE comprises all studies reporting rates of PE and DVT, the analysis of PE comprises all studies reporting PE as a separate outcome and the analysis of DVT comprises studies reporting DVT rates separately. Studies with a suspected high risk of bias have been excluded from these analyses.

Abbreviations: DVT, deep vein thrombosis; ICU, intensive care unit; PE, pulmonary embolism; VTE, venous thromboembolism.

^a^ In one study screening was performed in 28% of total patients (ICU, 51%; non‐ICU hospitalized, 14%).^15^

^b^ No screening for pulmonary embolism was performed.

^c^ All patients who were hospitalized at study baseline, excluding ICU patients. ICU admission during later hospital course was possible.

### 3.5 Characteristics of patients with VTE versus those without VTE

Available baseline characteristics of patients with VTE compared to those without VTE were aggregated and differences were analyzed within a random‐effect meta‐analysis (Table 4). Mean weighted age of patients with VTE and patients without VTE was similar, with a mean age of 63.3 years (SD, 3.9) and 63.4 years (SD, 2.8), respectively. Men were 1.5 times more likely to develop VTE (95% CI; 1.22‐1.72), while comorbidities did not differ between the two groups.

**Table 4:** Characteristics of patients with COVID‐19 with versus those without venous thromboembolism

	No./Total (%) of VTE patients	No./Total (%) of non‐VTE patients	Pooled OR for VTE (95%CI)	*P value*
Mean age (±SD) in years	**63.3** (±3.9)	**63.4** (±2.8)	…	…
Sex				
Male	627/940 **(66.7)**	2315/3803 **(60.9)**	1.45 (1.22‐1.72)	<.001
Female	313/940 **(33.3)**	1488/3803 **(39.1)**	Ref.	Ref.
Hypertension	278/584 **(47.6)**	1115/2359 **(47.3)**	0.88 (0.51‐1.51)	.65
Diabetes mellitus type 2	189/652 **(29.0)**	618/2725 **(22.7)**	0.97 (0.58‐1.63)	.92
Current or former smoker	75/446 **(16.8)**	296/1913 **(15.5)**	0.83 (0.42‐1.64)	.59
Cancer	58/676 **(8.6)**	306/2852 **(10.7)**	1.17 (0.72‐1.88)	.42
Chronic kidney disease	32/444 **(7.2)**	202/1914 **(10.6)**	0.76 (0.49‐1.19)	.23
Coronary artery disease	32/285 **(11.2)**	190/1731 **(11.0)**	1.04 (0.67‐1.60)	.87
Congestive heart failure	25/389 **(6.4)**	161/2025 **(8.0)**	0.86 (0.51‐1.46)	.58
Chronic lung disease	49/424 **(11.6)**	179/2101 **(8.5)**	0.92 (0.49‐1.70)	.78
Prior VTE	38/524 **(7.3)**	132/2128 **(6.2)**	1.61 (0.97‐2.67)	.07
Cardiovascular disease	42/121 **(34.7)**	72/404 **(17.8)**	1.52 (0.51‐4.56)	.46
Immune disease or immunosuppression	11/252 **(4.4)**	98/1310 **(7.5)**	1.24 (0.60‐2.59)	.56
Cerebrovascular disease	18/161 **(11.2)**	67/1273 **(5.3)**	0.54 (0.22‐1.33)	.18

Abbreviations: CI, confidence interval; OR, odds ratio; VTE, venous thromboembolism.

### 3.6 d‐dimer and the risk of VTE

**Figure 3:**
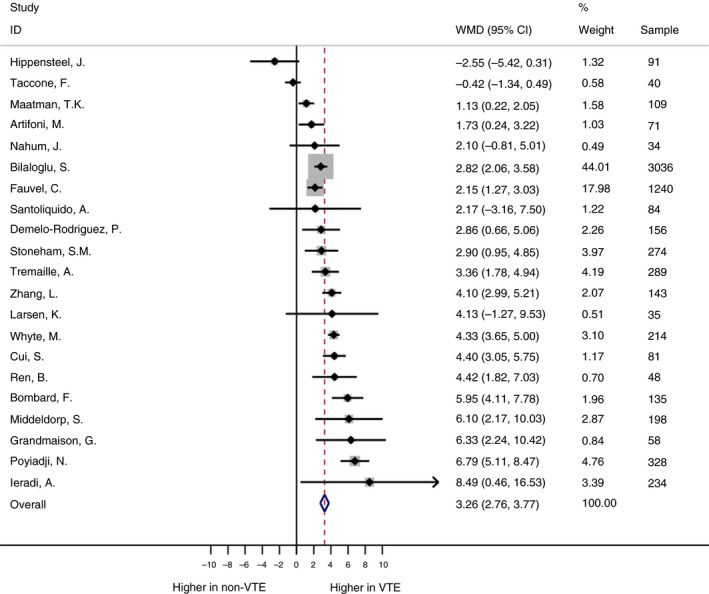
Differences in baseline d‐dimer between patients with VTE and patients without VTE. Patients developing VTE had higher baseline d‐dimer levels compared to those without VTE. D‐dimer levels at baseline were available in 21 studies, including 6633 patients. In the pooled analysis, levels of d‐dimer were substantially higher at baseline in patients experiencing VTE (WMD, 3.26 µg/mL (95% CI, 2.76‐3.77; *P* < .001; I^2^, 87.3%). CI, confidence interval; VTE, venous thromboembolism; WMD, weighted mean difference

d‐dimer levels at baseline were available in 21 studies, including 6633 patients. Patients developing VTE had higher baseline d‐dimer levels compared to those without VTE (weighted mean d‐dimer levels, 5.18 µg/mL [SD, 2.59] vs 1.13 µg/mL [SD, 0.95]) with a WMD of 3.26 µg/mL (95% CI, 2.76‐3.77; *P* < .001; I^2^, 87.3%) (Figure 3).

## 4 DISCUSSION

In this systematic review and meta‐analysis, data from studies reporting on rates of VTE in patients with COVID‐19 were aggregated to estimate the prevalence of VTE. We found that the burden of VTE associated with COVID‐19 is substantial, with an overall VTE prevalence estimate of 14.1% across all identified studies. However, rates of VTE varied across different health care settings (ICU vs non‐ICU hospitalized patients), depending on whether systematic screening was performed and on outcome definitions in the selected studies. In subgroup analysis, rates of VTE ranged from 5.5% in non‐ICU hospitalized patients without ultrasound screening to 45.6% in ICU patients undergoing screening strategies. Since no PE screening was performed, the PE prevalence of 3.5% in non‐ICU hospitalized patients and 13.7% in ICU patients might provide a robust estimate and strongly highlights the high risk of VTE in patients with COVID‐19, especially in those requiring intensive medical care.

It is known from large clinical trials in critically ill patients with various underlying diseases that the rate of VTE in the ICU setting is elevated, with VTE rates ranging from 5% to 15%.^82^, ^83^, ^84^, ^85^, ^86^ Higher VTE rates in patients with COVID‐19 in the ICU and also non‐ICU setting might not only be explained by hospitalization and complications occurring during the course of the disease such as systemic inflammatory response syndrome, acute respiratory distress syndrome, shock, and organ failure but support the hypothesis of direct involvement of the viral infection with effects on the vascular and hemostatic system leading to a prothrombotic state and high risk of VTE. Interestingly, a small study of critically ill patients with severe acute respiratory syndrome coronavirus from the early 2000s reported similarly high VTE rates (14 of 46 patients suffered from VTE).^87^ VTE events were observed less frequently in other respiratory viruses such as the Middle East respiratory syndrome coronavirus (coagulopathy was reported in 2 of 161 hospitalized patients) ^88^ and influenza viruses (4 of 119 hospitalized patients developed VTE). ^89^ Taken together, the increased risk of VTE in patients with COVID‐19 appears to be substantial, and while the mechanisms are not yet understood, similar rates in severe acute respiratory syndrome and COVID‐19 in contrast to Middle East respiratory syndrome and influenza might speculatively suggest a common underlying pathophysiology.

Interestingly, autopsy studies in patients with COVID‐19 revealed severe endothelial injury, endotheliitis, increased angiogenesis, and widespread vascular thrombosis with microangiopathy and occlusion of alveolar capillaries.^1^, ^2^, ^90^, ^91^, ^92^ Based on such findings, the etiology of the increased PE rates reported in patients with COVID‐19 has been discussed, and two not mutually exclusive pathomechanisms have been proposed. On the one hand, it has been suggested that in situ pulmonary thrombi, which develop on the basis of diffuse alveolar and local vascular damage, microangiopathy, and inflammation in the pulmonary circulation triggered by the virus rather than “classical” PE itself, may contribute to the high prevalence of PE observed in patients with COVID‐19.^93^, ^94^, ^95^, ^96^, ^97^ On the other hand, DVT rates of up to 90% in studies, where ultrasound screening was performed in ICU patients, support the hypothesis of embolism originating from peripheral thrombosis rather than pulmonary in situ thrombosis largely contributes to the substantial burden of pulmonary artery occlusion observed in patients with COVID‐19. However, the exact role, data on frequency, and clinical consequences of in‐situ pulmonary thrombosis in COVID‐19 need further investigations.

We believe that our meta‐analysis is representative of patients with COVID‐19 requiring hospitalization, as our systematic review confirmed the previously reported sex differences in patients with COVID‐19 (higher proportion of men among more severe disease).^98^ The sex differences further increased among patients admitted to the ICU, suggesting that men were more likely to suffer from greater disease severity than women.^99^ Correspondingly, men were at higher risk to develop VTE, but we observed no association between comorbidities and risk of VTE. Interestingly, age did not differ between the groups. This suggests that in contrast to the general population, age did not contribute to the VTE risk in patients with COVID‐19.^100^ Similar results have been reported for VTE risk in patients with cancer, suggesting that the high VTE baseline risk of the underlying disease overwhelms general risk factors such as age.^101^ Furthermore, explorative analysis has revealed that d‐dimer levels were higher in patients developing VTE compared to those who remained free from a VTE event.

Our findings support guidance statements from experts and scientific societies, which suggest that thromboprophylaxis is a key element in the medical care of patients with COVID‐19, especially in those with severe illness.^7^, ^8^, ^102^, ^103^, ^104^ However, VTE occurred in many patients despite the use of thromboprophylaxis, and even patients with therapeutic anticoagulation developed VTE. Therefore, the ideal anticoagulation approach to reduce the high risk of VTE in patients with COVID‐19 needs to be established. Further, the observed higher baseline d‐dimer levels in patients who had VTE strengthens the idea that d‐dimer–guided thromboprophylaxis strategies should be evaluated in prospective randomized controlled trials.

The main limitation of our meta‐analysis is the high heterogeneity of included studies with regard to design, clinical setting, local practice (eg, with respect to thromboprophylaxis strategies), and consequently highly variable event rates. Additionally, the disproportionate number of ICU studies with higher VTE rates than the general ward population may confound the overall estimation of VTE prevalence in patients with COVID‐19. To address this issue, we aimed at thoroughly describing the respective clinical settings and provide subgroup analysis, for example, ICU versus non‐ICU hospitalized patients or according to diagnostic approaches (studies with screening vs no screening for DVT) to provide a more precise estimate of VTE rates. Further, early reports of high VTE rates in patients with COVID‐19 might have led to the implementation of more specific and intensive thromboprophylaxis approaches over time, which might have confounded the outcomes in subsequently conducted studies. We have analyzed studies according to the date of the last patient recruitment, and visual inspection reveals a decrease of VTE rates of reported studies over time (Figure S2). We also provided data on thromboprophylaxis modalities for the respective studies to allow a better interpretation of differences observed in the studies. However, the generalizability of the results of our systematic review and meta‐analysis still needs to be interpreted with caution because only data from patients in North America, Europe, and Asia were available and included in the meta‐analysis. Upon visual inspection, VTE rates across continents and countries seem to be mainly related to between‐study heterogeneity with respect to study design, clinical setting, and local clinical practice with regard to thromboprophylaxis (Figure S4).

Given the high mortality, especially in ICU patients with COVID‐19, competing risk of death might lead to an underdiagnosis of VTE. Further, the concern of restricting the use of imaging to avoid disease exposure to health care workers might further lead to false low rates of VTE in patients with COVID‐19. These uncontrollable factors in a study‐level analysis should be considered upon interpreting and generalizing our findings. Also, the practice of avoiding imaging due to concerns about health care worker exposure should be critically reviewed given the risk of underdiagnosis and consequently undertreatment of patients.

Furthermore, exploratory analysis of d‐dimer levels between patients who developed VTE and those who did not is limited by the lack of patient‐level data and the inability to adjust for between‐assay variability. Therefore, this exploration should be interpreted with appropriate caution and regarded as hypothesis generating.

Finally, there is some evidence that nonhospitalized patients with COVID‐19 are at increased risk of developing VTE as well. ^105^ Because of the unavailability of sufficient data within our meta‐analysis, we were unable to provide prevalence estimates for this population of patients, and our findings are therefore not representative for the outpatient setting of COVID‐19.

In summary, we found a high prevalence of VTE in patients with COVID‐19 in hospitalized non‐ICU patients, and especially high VTE rates in those being critically ill and requiring intensive medical care. There is a clinical need for further research to better understand the risk and prevent VTE in patients with COVID‐19. These findings support the broad use of thromboprophylaxis, specifically in ICU patients. Future randomized clinical trials are needed to assess whether patients with COVID‐19 may benefit from an intensified anticoagulation approach compared to standard thromboprophylaxis or whether a biomarker‐based personalized thromboprophylaxis regimen reduces the high prevalence of VTE in patients with COVID‐19.

## AUTHOR CONTRIBUTIONS

SN and FM contributed to study design, data collection, data interpretation, statistical analysis, and drafting of the manuscript. CA contributed to study design, data interpretation, and critical review of the manuscript. IP contributed to data interpretation and critical review of the manuscript. SN, FM, and CA are the guarantor of this work and, as such, had full access to all the data in the study and take responsibility for the integrity of the data and the accuracy of the data analysis. All authors read the manuscript and approved its submission.

## RELATIONSHIP DISCLOSURE

SN and FM report no potential conflict of interest. BJ has received reimbursement for scientific advice from Bayer, Octapharma, and Sanofi. The ongoing ACOVACT trial randomizing patients with COVID to different anticoagulants is financially supported by the Austrian Federal Ministry of Education, Science and Research. IP received honoraria for occasional lectures and advisory board meetings from Bayer, Daiichi‐Sanchyo, Pfizer, and Sanofi. CA received honoraria for lectures and participation in advisory board meetings from Bayer, Daiichi‐Sanchyo, Pfizer/BMS, Sanofi, Shire/Takeda, Sobi, and CSL Behring.

## Supporting information

Supplementary Material
