# Supplementary material for: Risk of venous thromboembolism in patients with COVID‐19: A systematic review and meta‐analysis
Source: Res Pract Thromb Haemost. 2020 Oct 13;4(7):1178–91. doi: 10.1002/rth2.12439 (PMC7537137; doi:10.1002/rth2.12439)
Supplement: Supplementary file 1 — Supplementary Material [file RTH2-4-1178-s001.docx]

**Risk of venous thromboembolism in patients with COVID-19: A systematic review and meta-analysis – Supplemental Data**

# Supplementary Methods

## Search strategy

We conducted a systematic search of the literature using EMBASE, MEDLINE, and the WHO COVID-19 research database.

Search terms for literature review were predefined and included:

- COVID-19, SARS-CoV2, 2019 novel coronavirus disease, 2019 novel coronavirus infection, 2019-nCoV disease, 2019-nCoV infection, coronavirus disease 2019, coronavirus disease-19

- venous thromboembolism, venous thrombosis, deep vein thrombosis, pulmonary embolism

## Search protocol

MEDLINE (via PubMed):

(COVID-19*[tiab] OR SARS-CoV-2*[tiab] OR 2019 novel coronavirus*[tiab] OR [tiab] OR 2019-nCoV*[tiab] OR coronavirus disease 2019*[tiab] OR coronavirus disease-19*[tiab] OR COVID*[tiab]) OR coronavirus*[tiab] OR nCoV*[tiab])

AND

((venous thromboembolism[mesh] OR venous thrombosis[mesh] OR pulmonary embolism[mesh]) OR (thrombo*[tiab] OR venous thromb*[tiab] OR VTE[tiab] OR pulmonary embol*[tiab] OR DVT[tiab] OR PE[tiab]))

Years: 2019 and 2020

EMBASE:

#1 'COVID-19'/exp

#2 ' COVID-19*':ti,ab OR 'SARS-CoV-2*’:ti,ab OR '2019 novel coronavirus*’:ti,ab OR '2019 novel coronavirus*’:ti,ab OR '2019-nCoV*’:ti,ab OR 'coronavirus disease 2019*’:ti,ab OR 'coronavirus disease-19*’:ti,ab OR 'COVID*’:ti,ab OR 'coronavirus*’:ti,ab OR 'nCoV*’:ti,ab

#3 'venous thromboembolism’/exp

#4 'venous thrombosis’/exp

#5 'pulmonary embolism’/exp

#6 'thrombo*’:ti,ab OR 'venous thromb*’:ti,ab OR 'VTE*’:ti,ab OR 'pulmonary embol*’:ti,ab OR 'DVT’:ti,ab OR 'PE’:ti,ab

#7 (#1 OR #2) AND (#3 OR #4 OR #5 OR #6)

Filter:

#8 #7 AND 'human'/de

WHO COVID-19 research database

(tw:(venous thromboembolism)) OR (tw:(venous thrombosis)) OR (tw:(pulmonary embolism)) OR (tw:(thrombo*)) OR (tw:(venous thromb*)) OR (tw:(pulmonary embol*)) OR (tw:( DVT)) OR (tw:( PE)) OR (tw:( embol*))

## Data extraction

- Study identifiers: first author, publication year, title, country

- Study specific methodological data: sample size, study design, single vs multicenter, health care setting (ambulatory, non-ICU hospitalized, ICU), ultrasound screening strategy, follow-up time, in- and exclusion criteria,

- Patient and disease specific data: sex, age, BMI, comorbidities, hospitalization, ICU-admission, disease severity, thromboprophylaxis

- Outcome specific data: Venous thromboembolism (VTE) (overall, deep vein thrombosis (DVT), pulmonary embolism (PE), catheter-related thrombosis), mortality

## Risk of bias evaluation

The Joanna Briggs Institute Critical Appraisal Checklist(1) was used to evaluate risk of bias of included studies. The 10 criteria used to assess the methodological quality of studies reporting prevalence data are summarised below. Questions may be answered as follows: yes, no, unclear, or not applicable.

| **Criteria** | **Y** | **N** | **U** | **NA** |
| --- | --- | --- | --- | --- |
| 1. Was the study sample representative of the target population? |  |  |  |  |
| 1. Where study participants recruited in an appropriate way? |  |  |  |  |
| 1. Was the sample size adequate? |  |  |  |  |
| 1. Where the study subjects and the study setting described in detail? |  |  |  |  |
| 1. Was the data analysis conducted with sufficient coverage of the identified population? |  |  |  |  |
| 1. Were objective, standard criteria used to measure the condition of interest? |  |  |  |  |
| 1. Was the condition measured reliably? |  |  |  |  |
| 1. Was there appropriate statistical analysis? |  |  |  |  |
| 1. Were all important confounding factors/subgroups/differences identified and accounted for? |  |  |  |  |
| 1. Were subpopulations identified using objective criteria? |  |  |  |  |

Y=yes; N=no; U=unknown; NA=not applicable

# Supplementary Figures

## Figure S1A&B: Funnel Plots with 95% pseudo confidence intervals


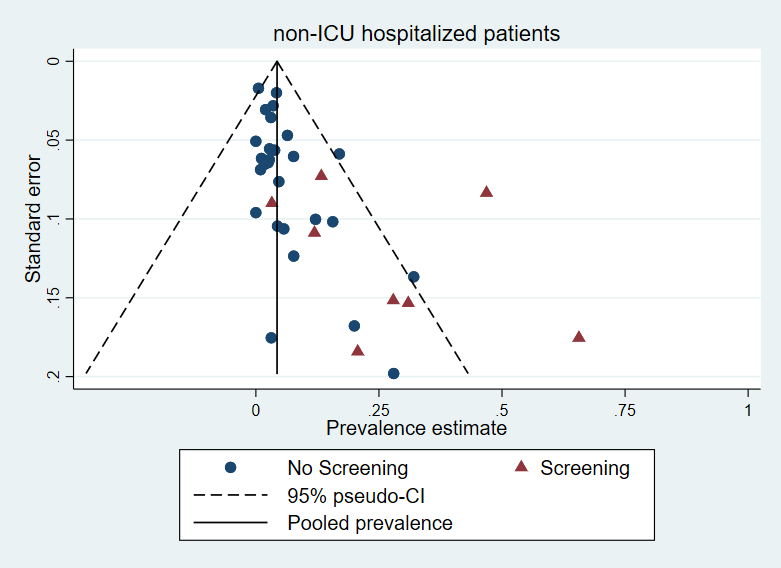

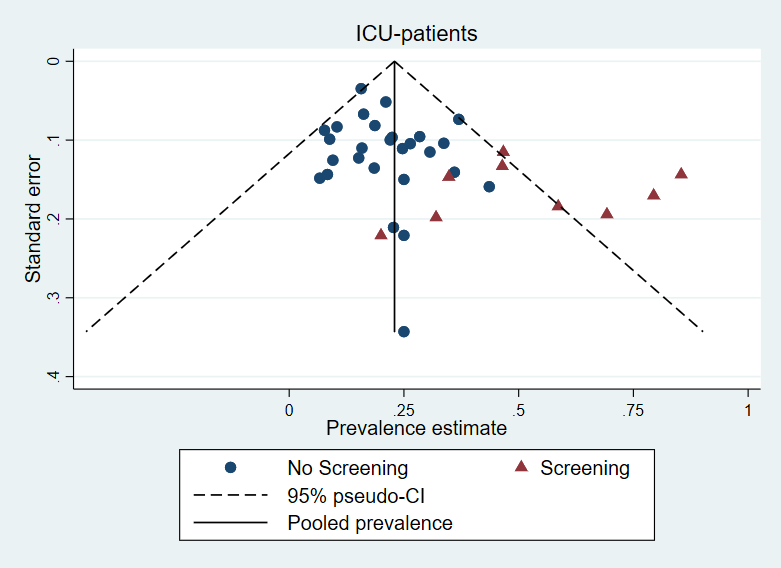


Upon visual inspection of the Funnel plots, no indication for publication bias was detected, with outliers in the distribution being explained by differences in ultrasound screening strategies. Figure S1A shows VTE prevalence estimates and its standard error of studies reporting on non-ICU hospitalized patients**,** while Figure S1B displays the same for studies on ICU patients.

Abb.: CI, confidence interval; ICU, intensive care unit

## Figure S2: Bubble plot: Rate of VTE plotted against publication date


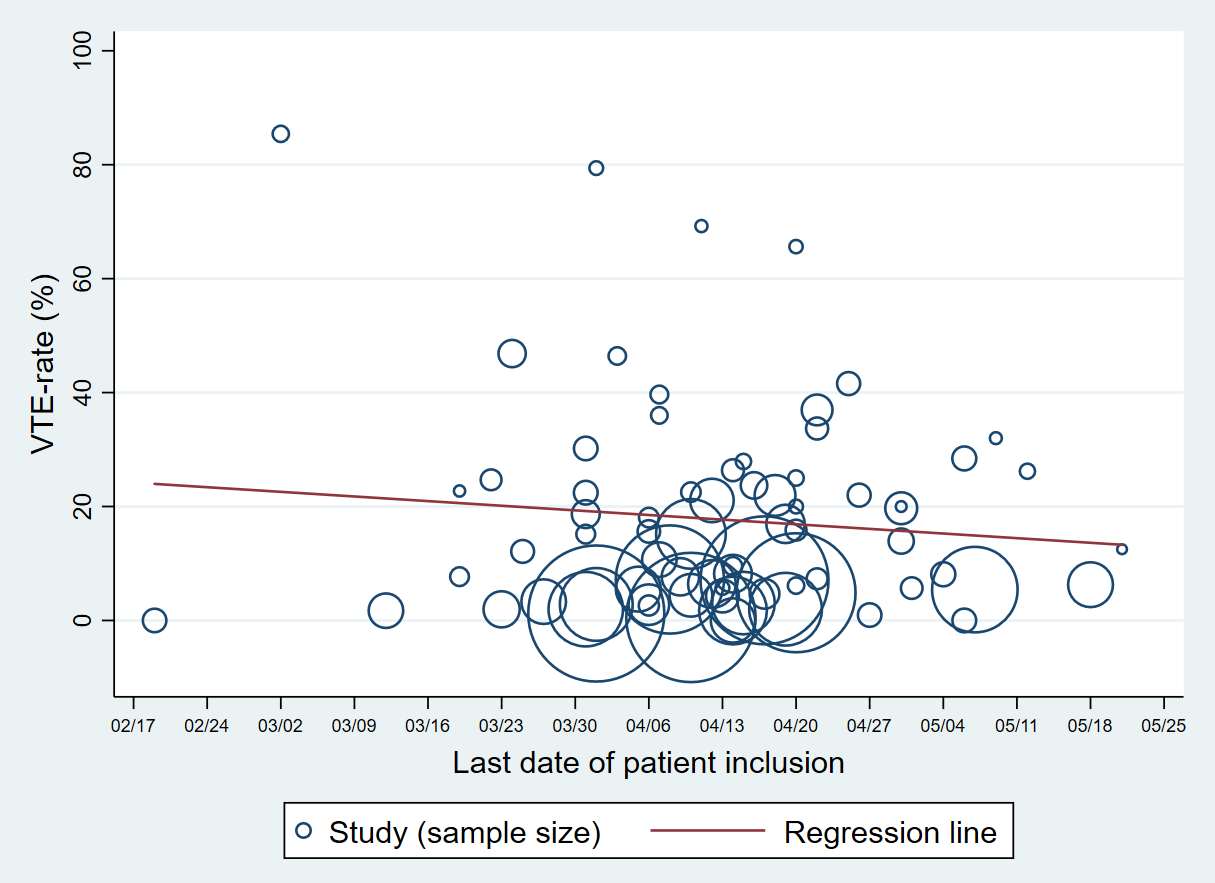


Figure S2 shows a decrease in VTE rates over time. Each bubble represents one study, while the bubble size represents the sample size. On the time-axis the last recruitment date of each respective study is displayed.

Abb.: VTE, venous thromboembolism

## Figure S3A: Forrest plots of VTE, PE and DVT in ICU patients


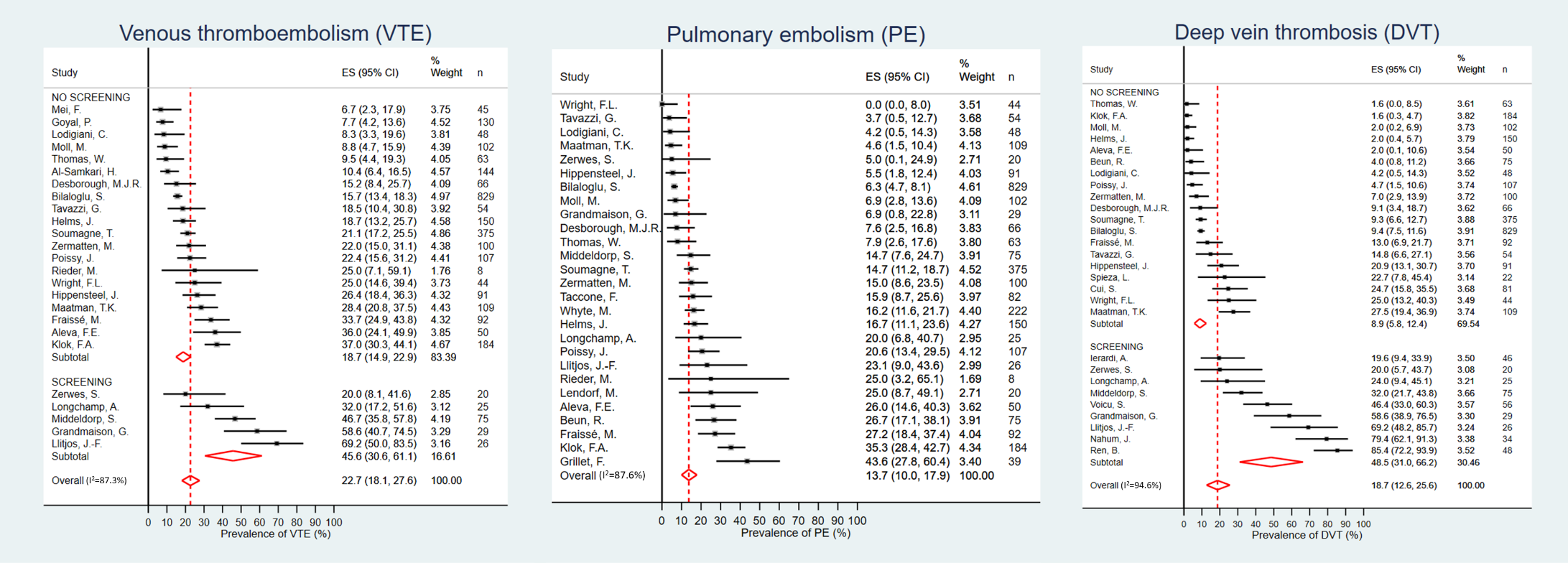


## Figure S3B: Forrest plots of VTE, PE and DVT in non-ICU hospitalized patients


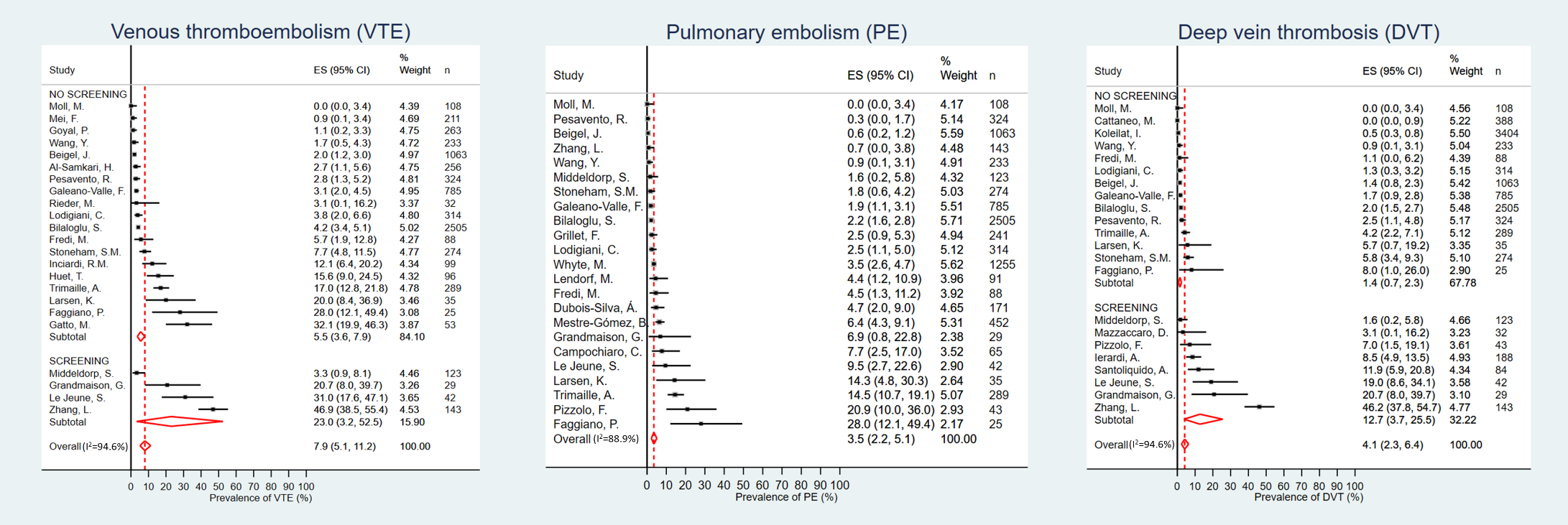


Subgroup analysis on the prevalence of venous thromboembolism, pulmonary embolism, and deep vein thrombosis in patients treated an intensive care unit or on the general ward (non-ICU hospitalized patients). No screening in PE patients was performed.

Abb.: CI, confidence interval; DVT, deep vein thrombosis; ES, prevalence estimate; PE, pulmonary embolism; VTE, venous thromboembolism

.

## Figure S4: Geographical differences in VTE prevalence


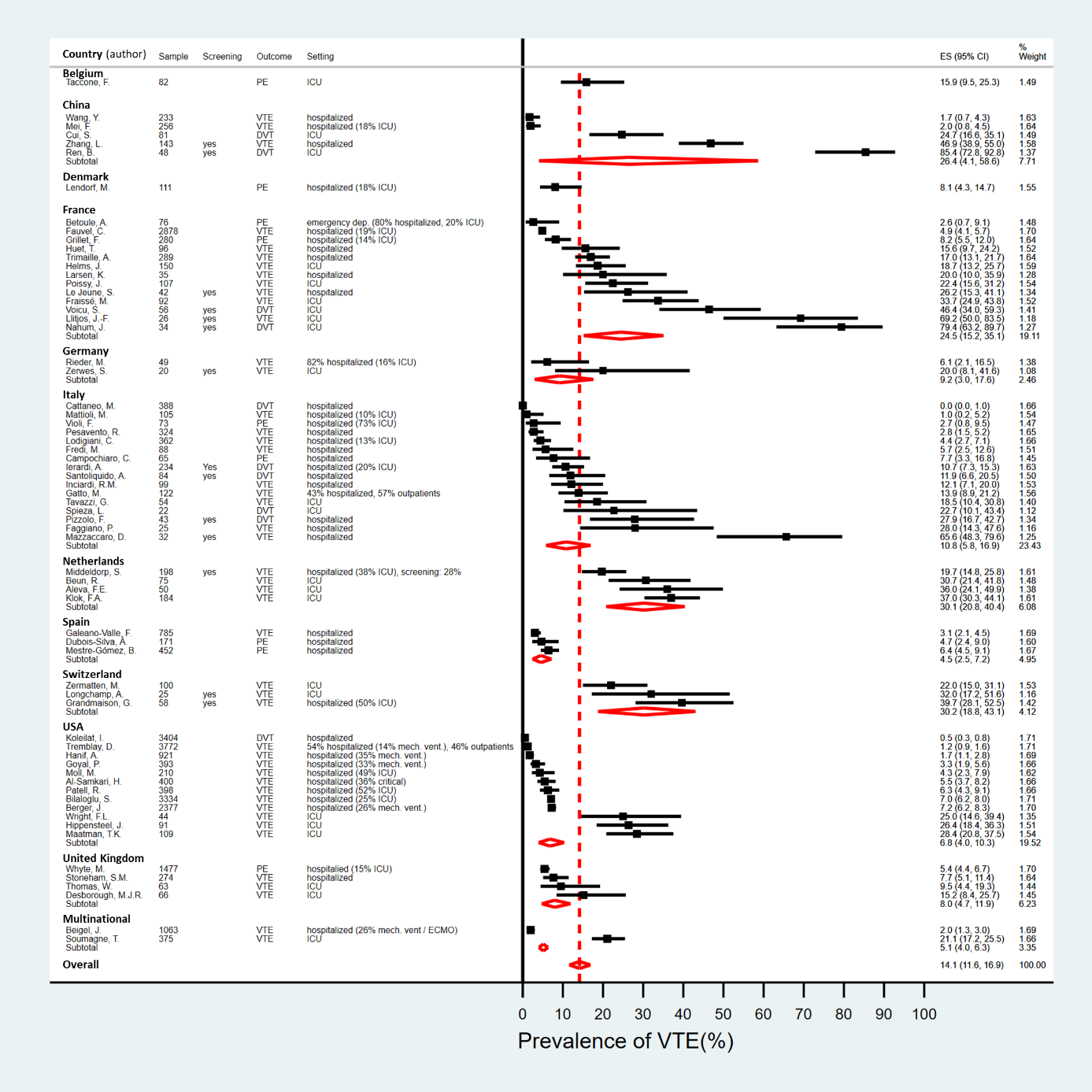


Figure S4 shows exploration of geographical differences of rates of venous thromboembolism (VTE). VTE rates across different countries vary. However, this seems to be mainly explained by the different study design, clinical setting, and if DVT screening was performed.

Abb.: DVT, deep vein thrombosis; ECMO, extracorporal membrane oxygenation; ES, prevalence estimate; ICU, intensive care unit; mech. vent., mechanically ventilated patients; PE, pulmonary embolism; VTE, venous thromboembolism. .

# Supplementary Tables

## Table S1 Study design and setting

| **Author** | **Country** | **Sample Size** | **Study Design** | **Health care setting** | **Ultra-sound screening** | **Follow-up** | **Exclusion criteria** | **Outcome definition** | **Anti-coagulation** |
| --- | --- | --- | --- | --- | --- | --- | --- | --- | --- |
| Maatman, T.K. (2) | USA | 109 | multicenter retrospective cohort study | ICU | no | Not defined, discharged from ICU: 13 days (IQR, 9–17 days; 7 patients (6%) still in ICU) | <18 years, pregnant, comfort care only | primary outcome, VTE radiological confirmed | 94% prophylactic  6% therapeutic |
| Desborough, M.J.R. (3) | United Kingdom | 66 | single-center retrospective cohort study | ICU | no | Median duration of ICU days 9 ( IQR, 3-15), max follow-up 28 days after ICU admission | None | primary outcome, VTE radiological confirmed | 83% prophylactic, 17% therapeutic |
| Galeano-Valle, F. (4) | Spain | 785 | single-center prospective cohort study | hospitalized | no | Median days of hospitalization 24 (IQR, 15-27.2) | <18 years, known prior antiphospholipid antibodies assessment | primary outcome, VTE radiological confirmed | All but 5 (21%) VTE patients received thromboprophylaxis |
| Inciardi, R.M. (5) | Italy | 99 | single-center retrospective cohort study | hospitalized | n.r. | Length of stay mean 11.4 (SD 6.5) days | none | secondary outcome, VTE not defined | Not routinely administered |
| Artifoni, M. (6) | France | 71 | multicenter retrospective cohort study | hospitalized, ultrasound available | yes | Sonography performed in median (IQR) after admission: 13 (11–17) days | <18 years, therapeutic anticoagulation prior to admission, contraindication to anticoagulation | primary outcome, VTE radiological confirmed | 99% prophylactic |
| Wang, Y. (7) | China | 233 | multicenter randomized controlled trial | Hospitalized | n.r. | Duration of hospital stay median 25 (IQR, 16-38) in treatment group and 24 (IQR, 18 to 36) in placebo group | <18 years, symptom onset ≥ 12 days, oxygen saturation ≥ 94% or Horovitz-index ≥ 300 mmHG | Adverse event, VTE not defined | n.r. |
| Ren, B. (8) | China | 48 | multicenter cross sectional study | ICU | yes | Interval between ICU admission and DVT diagnosis 12 (IQR 8-14) days | prior DVT, recent surgery | primary outcome, DVT radiological confirmed | 98% prophylactic |
| Demelo-Rodríguez, P. (9) | Spain | 156 | single-center prospective cohort study | hospitalized | yes | Median days of hospitalization until ultrasound 9 days (IQR 5-17) | D-dimer <1000 ng/ml, <18y, hospitalized less than 48h; therapeutic anticoagulation, prior DVT, clinical signs/symptoms for DVT at inclusion | primary outcome, DVT radiological confirmed | 98% prophylactic |
| Helms, J. (10) | France | 150 | multicenter prospective cohort study | ICU | no | Median length of ICU stay: 9.6 ± 4.2 | none | primary outcome, VTE radiological confirmed | 70% therapeutic, 30% prophylactic |
| Llitjos, J.-F.(11) | France | 26 | multicenter retrospective cohort study | ICU | yes | n.r. | none | primary outcome, VTE radiological confirmed | 31% prophylactic, 69% therapeutic |
| Stoneham, S.M. (12) | United Kingdom | 274 | multicenter retrospective cohort study | hospitalized | no | n.r. | none | primary outcome, VTE radiological confirmed | n.r. |
| Wright, F.L. (13) | USA | 44 | single-center retrospective cohort study | ICU | no | Admission to VTE in median 12 (IQR 7-14.5) days | Patients without thromboelastography-testing were excluded, but every eligible patients had thromboelastography testing | primary outcome, VTE radiological confirmed | 100% prophylactic |
| Louhaichi, S. (14) | Tunisia | 20 | single-center retrospective cohort study | hospitalized | n.r. | n.r. | n.r. | outcome not defined | n.r. |
| Thomas, W. (15) | United Kingdom | 63 | single-center retrospective cohort study | ICU | no | Median number of days 8 (range 1–28) | none | primary outcome, VTE radiological confirmed | 100% prophylactic |
| Bombard, F. (16) | France | 135 | single-center retrospective cross sectional study | 53% hospitalized (18% ICU), 47% outpatients; CTPA | no | n.r. | Only patients with CTPA were included | primary outcome, PE radiological confirmed | 100% prophylactic |
| Faggiano, P. (17) | Italy | 25 | single-center retrospective cohort study | hospitalized | no | n.r. | none | primary outcome, VTE radiological confirmed | 20% prophylactic, 16% therapeutic |
| Klok, F.A. (18, 19) | Nether-lands | 184 | multicenter retrospective/prospective cohort study | ICU | no | Median 14 days (IQR 6-19) | none | primary outcome, VTE radiological confirmed | 91% prophylactic, 9% therapeutic |
| Middeldorp, S. (20) | Netherlands | 198 | single-center retrospective cohort study | hospitalized (38% ICU) | yes | Median: 7 days (IQR 3-13) | Patients diagnosed with COVID-19 during hospital stay for other medical condition | primary outcome, VTE radiological confirmed | 84% prophylactic (dose changed during study),  10% therapeutic |
| Tavazzi, G. (21) | Italy | 54 | single-center prospective cohort study | ICU | n.r. | n.r. | none | primary outcome, VTE radiological confirmed | 100% prophylactic |
| Beun, R. (22) | Netherlands | 75 | single-center retrospective cohort study | ICU | no | n.r. | n.r. | primary outcome, VTE radiological confirmed | n.r. |
| Cui, S. (23) | China | 81 | single-center retrospective cohort study | ICU | no | n.r. | n.r. | primary outcome, DVT radiological confirmed | 0% prophylactic |
| Spieza, L. (24) | Italy | 22 | single-center prospective case-control study | ICU | no | n.r. | Known preexisting congenital bleeding, thrombotic disorders, preexisting acquired coagulopathy, active cancer, ongoing anticoagulation therapy, pregnancy | Secondary, outcome, DVT not defined | Likely 100% prophylactic |
| Al-Samkari, H. (25) | USA | 400 | multicenter retrospective cohort study | hospitalized (36% critically ill) | no | 3226 patient-days = mean of 8 days | <18 years | primary outcome, VTE clinically suspected and radiological confirmed | n.r. |
| Fraissé, M. (26) | France | 92 | single-center retrospective cohort study | ICU | no | Time to thromboembolic event: median 9 (IQR, 3-21) days on ICU | none | Primary outcome, catheter-related thrombosis excluded | 47% prophylactic, 53% therapeutic |
| Hippensteel, J. (27) | USA | 91 | single-center retrospective cohort study | ICU | no | Average hospital length of stay for VTE and non-VTE patients: 26 vs 16 days | < 18 years, therapeutic anticoagulation, ECMO | Primary outcome, VTE radiological confirmed | n.r. 54% received therapeutic during stay |
| Voicu, S. (28) | France | 56 | single-center prospective cohort | ICU | yes | First sonography performed 3 (2-4) days post-intubation, in 30% second sonography was performed 8 (5-9) days post-intubation | prior VTE | Primary outcome, DVT radiological confirmed | 87% prophylactic, 13% therapeutic |
| Guo, T. (29) | China | 105 | multicenter retrospective cohort study | hospitalized  (10.5% critically ill) | no | Median 17 (IQR, 13-23) days in-hospital | <60 years | Secondary outcome, VTE not defined | n.r. |
| Campochiaro, C. (30) | Italy | 65 | single-center retrospective cohort study | hospitalized | no | 28 days | several criteria unrelated to VTE | Adverse event, PE not defined | 100% prophylactic |
| Zhang, L. (31) | China | 143 | single-center prospective cohort study | hospitalized | yes | Median time from admission to detection of DVT was 9 days  (IQR 6 -13 days) | <18 years | Primary outcome, VTE radiological confirmed | 37% prophylactic, 63% nonprophylaxis |
| Poyiadji, N. (32) | USA | 328 | multicenter retrospective cohort study | 25% ICU, CTPA | no | Length of stay 9 days (SD 8) | poor CT contrast or limited by motion | Primary outcome, PE radiological confirmed | n.r. |
| Criel, M. (33) | Belgium | 82 | single-center cross sectional study | hospitalized (37% ICU) | yes | Cross sectional study | none | Primary outcome, radiological confirmed insidious DVT | 65% prophylactic (non-ICU), 35% therapeutic (ICU) |
| Poissy, J. (34) | France | 107 | single-center retrospective cohort study | ICU | no | Median time from ICU  admission of 6 days (range 1 to 18 days) | none | Primary outcome, VTE radiological confirmed | 100% at least prophylactic |
| Lodigiani, C. (35) | Italy | 362 | single-center retrospective cohort study | hospitalized (13% ICU) | no | Median stay at general ward 4 days (IQR 3-6), ICU median 12 days (IQR 8-15) | <18 years | Primary outcome, VTE radiological confirmed | ICU patients: 97% prophylactic, 3% therapeutic  General ward: 62% prophylactic. 23% therapeutic |
| Grillet, F. (36) | France | 280 | single-center retrospective cohort study | hospitalized (14% ICU) | no | Interval between symptom onset and PE mean12 (SD, 6) | Only patients with CTPA were included | Primary outcome, PE radiological confirmed | n.r. |
| Leonard-Lorant, I. (37) | France | 106 | multicenter retrospective cohort study | 46% ICU, CTPA | no | Interval between symptom onset and PE median 14 (IQR, 11-18) | Only patients with CTPA were included | Primary outcome, PE radiological confirmed | 40% prophylactic, 7% therapeutic |
| Longchamp, A. (38) | Switzerland | 25 | single-center prospective cohort study | ICU | yes | Median time in ICU until VTE 3 (IQR, 2-7) days | none | Primary outcome, radiological confirmed | 92% prophylactic, 8% therapeutic |
| Goyal, P. (39) | USA | 393 | multicenter retrospective cohort study | hospitalized (33% mech. vent.) | no | n.r. | <18 years | Secondary outcome, VTE not defined | n.r. |
| Beigel, J. (40) | multiple | 1063 | multicenter randomized controlled trial | hospitalized (26% mech. vent / ECMO) | no | 28 days | Severe liver or renal dysfunction, pregnancy or breast-feeding | Adverse events, VTE not defined | n.r. |
| Tremblay, D. (41) | USA | 3772 | multicenter retrospective cohort study | 54% hospitalized (14% mech. vent.), 46% outpatients | n.r. | n.r. | <18 years | Secondary outcome, defined as overt thrombosis | Prior therapeutic anticoagulation in 6%, prophylaxis n.r. |
| Archer, J.E. (42) | multinational | 1115 | multicentre prospective cohort study | surgical patients | n.r. | Length of stay for group 1 median 13 (IQR, 5-28), group 2 median 16 (IQR, f7-28) days | none | Secondary outcome, PE not defined | n.r. |
| Huet, T. (43) | France | 96 | single-center prospective cohort study | hospitalized | n.r. | 7 days | Bedridden, end of life pts; ICU at inclusion; respiratory failure due to other diagnosis; | Adverse events, radiological confirmed VTE | 100% prophylaxis |
| Bowles, L. (44) | United Kingdom | 35 | single-center retrospective cohort study | hospitalized (60% ICU), prolonged APTT | n.r. | n.r. | Anticoagulation therapy prior to admission | Secondary outcome, VTE clinically suspected and radiological confirmed | n.r. |
| Nahum, J. (45) | France | 34 | single-center prospective cohort study | ICU | yes | n/a, cross-sectional study | none | Primary outcome, DVT radiological confirmed | 100% prophylaxis |
| Zerwes, S. (46) | Germany | 20 | single-center prospective cohort study | ICU | yes | Hospitalized for mean 24.4 (SD, 10) days | none | Primary outcome, VTE radiological confirmed | 100% at least prophylactic |
| Betoule, A. (47) | France | 76 | single-center prospective cohort study | emergency dep. (80% hospitalized, 19% ICU) | no | n.r. | n.r. | Primary outcome, PE not defined | n.r. |
| Gervaise, A. (48) | France | 72 | single-center prospective cohort study | emergency dep. (57% severe/critically ill), CTPA | no | Time from symptoms to CTPA mean 7.6 (SD, 4.2) | Only patients with CTPA were included | Primary outcome, PE radiological confirmed | n.r. |
| Grandmaison, G. (49) | Switzer-land | 58 | single-center cross sectional study | hospitalized (50% ICU) | yes | n/a, cross-sectional study | none | Primary outcome, VTE radiological confirmed | Reported for ICU |
| Le Jeune, S. (50) | France | 42 | single-center retrospective cohort study | hospitalized | yes | DVT screening median 4 (IQR, 2-6) days after admission | None | Primary outcome, VTE radiological confirmed | 83% prophylactic, 17% therapeutic |
| Taccone, F. (51) | Belgium | 82 | single-center retrospective cohort study | ICU | no | CTPA 7 (IQR, 4-8) days after admission | n.r | Primary outcome, PE radiological confirmed | 100% at least prophylactic |
| Violi, F. (52) | Italy | 73 | single-center obervational study | hospitalized (73% ICU) | no | hospital stay in median 15 (IQR, 10-22) days | <18y | Primary outcome, PE not defined | 100% at least prophylactic |
| Blumfield, E. (53) | USA | 16 | single-center retrospective cohort study | hospitalized (pediatric, MIS-C; 69% ICU) | no | n.r. | n.r. | Secondary outcome, PE radiological confirmed | n.r. |
| Santoliquido, A. (54) | Italy | 84 | single-center cohort study | hospitalized | yes | DVT screening 5.8 (SD, 2.2) days after admission | <18y, ICU-patients, full-dose anticoagulation | Primary outcome, DVT radiological confirmed | 100% prophalyctic |
| Mestre-Gómez, B. (55) | Spain | 452 | single-center retrospective cohort study | hospitalized | no | 18 (IQR, 12.5-22) days from COVID-19 symptoms to PE diagnosis | Only patients with CTPA were included | Primary outcome, PE radiological confirmed | 79% prophylactic |
| Patell, R. (56) | USA | 398 | single-center retrospective cohort study | hospitalized (52% ICU) | no | 28 days | <18y | Primary outcome, VTE radiological confirmed | 89% at least prophylactic |
| Berger, J. (57) | USA | 2377 | single-center retrospective cohort study | hospitalized, 38% critically ill | no | n.r. | Patients with no D-dimer value were excluded (n=405) | Secondary outcome, VTE clinically suspected and radiological confirmed | n.r. |
| Dubois-Silva, Á. (58) | Spain | 171 | single-center cross-sectional study | hospitalized | no | n.r. | ICU admission at enrolment, history of VTE within the past 3 months, and presence of a venous malformation | Primary outcome, PE radiological confirmed | n.r. |
| Fauvel, C. (59) | France | 2878 | multicenter retrospective cohort study | hospitalized (19% ICU) | no | Hospitalized for 6.8 (SD, 4.5) | Patients directly admitted to ICU | Primary outcome, VTE radiological confirmed | 71% prophylactic |
| Whyte, M. (60) | United Kingdom | 1477 | single-center retrospective cohort study | hospitalied (15% ICU) | no | n.r. | none | Primary outcome, PE radiological confirmed | n.r. |
| Rieder, M. (61) | Germany | 49 | single-center prospective cohort study | 82% hospitalized (16% ICU) | no | 30 days | Patients without 39 days of follow-up were excluded | Primary outcome, VTE radiological confirmed | n.r. |
| Mattioli, M. (62) | Italy | 105 | single-center retrospective cohort study | hospitalized (10% ICU) | no | 30 days | discharged, transferred in Intensive Care Unit (ICU), or dead within 48 h after admission | Primary outcome, VTE not defined | 100% at least prophylactic |
| Pesavento, R. (63) | Italy | 324 | single-center retrospective cohort study | hospitalized | no | hospital stay 12 (IQR, 8-18) days | patients requiring intubation for ventilatory support, intensive care or indefinite anticoagulation treatment | Secondary outcome, VTE radiological confirmed | 74% prophylactic, 26% higher dose |
| Bilaloglu, S. (64) | USA | 3334 | single-center retrospective cohort study | hospitalized (25% ICU) | no | n.r. | <18y | Primary outcome, VTE not defined | n.r. |
| Hanif, A. (65) | USA | 921 | single-center retrospective cohort study | hospitalized (35% intubated) | no | median stay 9 days | <18y | Primary outcome, VTE not defined | 97% at least prophylactic |
| Cho, E. (66) | USA | 158 | single-center retrospective cohort study | hospitalized | no | n.r. | < 18<, known DVT or PE | Primary outcome, DVT not defined | 90% prophylactic |
| Zermatten, M. (67) | Switzerland | 100 | single-center retrospective cohort study | ICU | no | n.r. | admitted to ICU for other reasons | Primary outcome, VTE radiological confirmed | 100% at least prophylactic |
| Moll, M. (68) | USA | 210 | single-center retrospective cohort study | hospitalized (49% ICU) | no | In median 7 (IQR, 4-14) days | none | Primary outcome, VTE radiological confirmed | 81% prophylactic 10% therapeutic |
| Chen, J. (69) | China | 1008 | single-center retrospective cohort study | hospitalized | no | n.r. | none | Primary outcome, PE radiological confirmed | n.r. |
| Trimaille, A. (70) | France | 289 | multicenter retrospective cohort study | hospitalized (non-critical) | no | length of stay 12.1 (SD, 7.5) days | none | Primary outcome, VTE not defined | 89% prophylactic |
| Naymagon, L. (71) | USA | 1065 | multicenter retrospective cohort study | hospitalized (ICU not known) | no | n.r. | no D-dimer level measurement | Secondary outcome, VTE radiological confirmed | 85% prophylactic, 13% therapeutic |
| Freund, Y. (72) | multinational | 974 | multicenter retrospective cohort study | underwent CTPA | no | n.r. | no CTPA performed | Primary outcome, PE radiological confirmed | n.r. |
| Lendorf, M. (73) | Denmark | 111 | single-center retrospective cohort study | hospitalized (18% ICU) | no | minimum 30 days | discharged <24h | Secondary outcome, PE not defined | n.r. |
| Marone, E. (74) | Italy | 101 | single-center retrospective cohort study | hospitalized | no | median time to DVT diagnosis 12 (range 4-20) days | patients without ultrasound | Primary outcome, DVT radiological confirmed | n.r. |
| Lachant, D. (75) | USA | 107 | single-center retrospective cohort study | hospitalized (39%) and ambulatory (61%) | no | n.r. | patients without anticoagulation treatment | Primary outcome, VTE radiological confirmed | 100% therapeutic |
| Koleilat, I. (76) | USA | 3404 | single-center retrospective case-control study | hospitalized (reported as admitted) | no | n.r. | Patients without ultrasound | Primary outcome, DVT radiological confirmed | n.r. |
| Fox, T. (77) | United Kingdom | 55 | single-center retrospective cohort study | 85% hospitalized 15% ambulatory | no | n.r. | <18 years, no haemoatological disorders | Secondary outcome, VTE clinically suspected and radiological confirmed | 58% prophylactic, 20% therapeutic |
| Mei, F. (78) | China | 256 | single-center retrospective cohort study | hospitalized (18% ICU) | no | n.r. | none | Primary outcome, VTE radiological confirmed | 100% pharmaceutical or mechanical prophylacxis |
| Aleva, F.E. (79) | Netherlands | 50 | single-center retrospective cohort study | ICU | no | 86 (82-94) days | none | Secondary outcome, VTE not defined | 100% at least prophylactic |
| Fredi, M. (80) | Italy | 88 | single-center retrospective case-control study | hospitalized | no | median 15 days (10-22) | n.r. | Secondary outcome, VTE not defined | n.r. |
| Pizzolo, F. (81) | Italy | 43 | single-center prospective cohort study | hospitalized (non-ICU) | yes | n.r. | therapeutic anticoagulation treatment | Primary outcome, DVT radiological confirmed | 100% prophylactic |
| Larsen, K. (82) | France | 35 | single-center retrospective cohort study | hospitalized | no |  | just returning travelers included | Primary outcome, VTE radiological confirmed | 80% prophylactic |
| Chen, S. (83) | China | 88 | single-center retrospective cohort study | ICU | yes | ICU stay median 22 ( IQR, 18-30) | patients with no thromboprophylaxis and no results on DVT screening | Primary outcome, DVT radiological confirmed | 100% prophylaxis |
| Ierardi, A. (84) | Italy | 234 | single-center prospective cohort study | hospitalized (20% ICU) | yes | n.r. | n.r. | Primary outcome, DVT radiological confirmed | 100% prophylaxis |
| Soumange, T. (85) | multinational | 375 | multicenter prospective cohort study | ICU | no | until day 28 | n.r. | Primary outcome, VTE radiological confirmed | 100% at least prophylactic |
| Gatto, M. (86) | Italy | 122 | multicenter retrospective case-control study | 44% hospitalized, 56% outpatient | no | n.r. | n.r. | Secondary outcome, VTE not defined | n.r. |
| Mazzaccaro, D. (87) | Italy | 32 | single-center prospective cohort study | hospitalized (non-ICU) | yes |  | n.r. | Primary outcome, VTE radiological confirmed | 100% at least prophylactic |
| Cattaneo, M. (88) | Italy | 388 | single-center retrospective cohort study | hospitalized (non-ICU) | no | days of in-hospital bed rest median 9 (IQR, 4-15) | none | Primary outcome, DVT radiological confirmed | n.r. |

Abb.: APTT, activated partial thromboplastin time; CTPA, computed tomography pulmonary angiogram; DVT, deep vein thrombosis; ECMO, extracorporal membrane oxygenation; ICU, intensive care unit; mech. vent., mechanically ventilated patients; n/a, not applicable; n.r., not reported; PE, pulmonary embolism; VTE, venous thromboembolism.

## Table S2: Patient demographics and outcomes

| **Author** | **Sample Size** | **Age**  **(mean / median)** | **Sex (% female)** | **BMI**  **(mean / median)** | **Comorbidities (%)** | **VTE**  **(%)** | **PE (%)** | **DVT (%)** | **VTE characteristics** | **Mortality (%)** |
| --- | --- | --- | --- | --- | --- | --- | --- | --- | --- | --- |
| Maatman, T.K. (2) | 109 | 61 | 43**%** | 34.8 | hypertension (68%), DM (39%), smoker (30%), COPD (16%), CHF (15%), CKD (15%) | 28.4 | 4.6 | 27.5 | 1 PE, 4 PE+DVT, 26 DVT; (5 CRT) | 25% |
| Desborough, M.J.R. (3) | 66 | 59 | 27**%** | 28 | hypertension (45%), DM (41%),  CKD (14%), cardiac disease (11%), chronic lung disease (9%), cancer (8%), previous VTE (8%) | 15.2 | 7.6 | 9.1 | 4 PE, 1 PE+DVT, 5 DVT; (6 CRT) | 30% |
| Galeano-Valle, F. (4) | 785 | 64 | 42**%** | 29.8 | prior VTE (8%), cancer (4%), | 3.1 | 1.9 | 1.7 | 11 PE, 4 PE+DVT, 9 DVT | n.r. |
| Inciardi, R.M. (5) | 99 | 67 | 19**%** | 18 | hypertension (64%), DM (31%), CHF (21%), smoker (20%), AF (19%), cancer (18%), CAD (16%), CKD (15%), COPD (9%) | 12.1 | - | - | Specifics n.r.; 3 fatal PE | 26% |
| Artifoni, M. (6) | 71 | 64 | 40**%** | 27.3 | hypertension (41%), DM (20%), smoker (9%), prior VTE (7%), cancer (6%) | 22.5 | 9.9 | 21.1 | 1 PE, 6 PE+DVT, 9 DVT | n.r. |
| Wang, Y. (7) | 233 | 66 | 44**%** | n.r. | hypertension (46%), DM (25%), CAD (9%) | 1.7 | 0.9 | 0.9 | 2 PE, 2 DVT | 14% |
| Ren, B. (8) | 48 | 70 | 46**%** | 33% ≥24 kg/m^2^ | hypertension (40%), DM (27%), CVD (23%), cerebrovascular disease (15%) | - | - | 85.4 | 41 DVT | 31% |
| Demelo-Rodríguez, P. (9) | 156 | 68 | 35**%** | 26.9 | cancer (10%) | - | - | 14.7 | 23 DVT | n.r. |
| Helms, J. (10) | 150 | 63 | 19**%** | n.r. | CVD (48%), DM (20%), cancer (6%), VTE (5%), cerebrovascular disease (5%), CKD (4%), immune disease (3%), chronic liver disease (3%) | 18.7 | 16.7 | 2.0 | 25 PE, 3 DVT | 8.7% |
| Llitjos, J.-F.(11) | 26 | 68 | 23**%** | n.r. | hypertension (85%), smoking (27%), cancer (0%) | 69.2 | 23.1 | 69.2 | 6 PE, 18 DVT | 12% |
| Stoneham, S.M. (12) | 274 | 67 | 33**%** | n.r. | DM (38%), pulmonary disease (38%), CVD (29%), CKD (19%), cancer (19%) | 7.7 | 1.8 | 5.8 | 5 PE, 16 DVT | 27.7% |
| Wright, F.L. (13) | 44 | 54 | 36**%** | 30 | hypertension (48%), , DM (41%), Asthma (9%), COPD (5%), arrythmia (7%), smoking (5%) | 25.0 | 0.0 | 25.0 | 0 PE, 11 DVT | n.r. |
| Louhaichi, S. (14) | 20 | 61 | 55**%** | n.r. | hypertension (55%), DM (30%), CAD (15%), CKD (5%), COPD (5%) | - | 10.0 | - | 2 PE | n.r. |
| Thomas, W. (15) | 63 | 49% between 50-69 | 31**%** | 19% ≥ 100kg | cancer (2%), prior VTE (2%) | 9.5 | 7.9 | 1.6 | 5 PE, 1 DVT; (1 CRT) | 16% |
| Bombard, F. (16) | 135 | 64 | 30**%** | n.r. | n.r. | - | 23.7 | - | 32 PE | n.r. |
| Faggiano, P. (17) | 25 | 71 | 28**%** | n.r. | CAD (52%), AF (32%), hypertension (32%), cancer (20%), CHF (16%), DM (8%) | 28.0 | 28.0 | 8.0 | 5 PE, 2 PE+DVT | 20% |
| Klok, F.A. (18, 19) | 184 | 64 | 24**%** | 87 kg | active cancer (3%) | 37.0 | 35.3 | 1.6 | 65 PE, 3 DVT | 22% |
| Middeldorp, S. (20) | 198 | 61 | 34**%** | 27 | prior vte (6%), cancer (4%) | 19.7 | 6.6 | 13.1 | 13 PE, 26 DVT | 19% |
| Tavazzi, G. (21) | 54 | 68 | 20**%** | 29.3 | n.r. | 18.5 | 3.7 | 14.8 | 2 PE, 8 DVT; (6 CRT) | n.r. |
| Beun, R. (22) | 75 | n.r. | n.r. |  | n.r. | - | 26.7 | 4.0 | 20 PE, 3 DVT | n.r. |
| Cui, S. (23) | 81 | 60 | 54**%** |  | smoking (43%), hypertension (25%), CAD (12%), DM (10%) | - | - | 24.7 | 20 DVT | 10% |
| Spieza, L. (24) | 22 | 67 | 9**%** | 30 | n.r. | - | - | 22.7 | 5 DVT | n.r. |
| Al-Samkari, H. (25) | 400 | 60 (non-critically ill), 65 (critical ill) | 43**%** | 41% ≥30 kg/m^2^ | non-critical vs critical ill: 32% vs 30% CVD, 21% vs 17% chronic lung disease, 25.4% vs 40% DM, 11.3% vs 14% immune compromise, 2% vs 4% CKD, 5% vs 4% chronic liver disease | 5.5 | 3.3 | 3.0 | 10 PE, 3 PE+DVT, 9 DVT | 7% |
| Fraissé, M. (26) | 92 | 61 | 21**%** | 30 | hypertension (64%), DM (38%), chronic respiratory disease (20%), CVD (10%), cerebrovascular disease (9%), CKD (8%), prior VTE (5%), AF (3%) | 33.7 | 27.2 | 23.0 | 19 PE, 6 PE+DVT, 6 DVT | 41% |
| Hippensteel, J. (27) | 91 | 56 | 42**%** | 32 | DM (33%), CVD (22%), chronic lung disease (19%), smoking (18%), CKD (12%), immunosuppressed (7%), cancer (3%) | 26.4 | 5.5 | 20.9 | 5 PE, 19 DVT | 43% |
| Voicu, S. (28) | 56 | n.r. | 25**%** | 30% reported as obese | hypertension (46%), DM (45%), ischemic heart disease (20%) | - | - | 46.4 | 26 DVT | n.r. |
| Guo, T. (29) | 105 | 67 | 54**%** | n.r. | hypertension (43%), DM (26%), cardiac disease (16%) | 0.0 | - | - | 0 VTE | 3% |
| Campochiaro, C. (30) | 65 | 62 | 14**%** | n.r. | hypertension (43%), DM (15%), CAD (15%), CKD (12%), COPD (5%), cancer (5%), smoking (3%) | - | 7.7 | - | 5 PE | 25% |
| Zhang, L. (31) | 143 | 63 | 48**%** | 23.6 | hypertension (39%), DM (18%), CAD (12%), smoking (6%), cancer (5%), CKD (3%) | - | 0.7 | 46.2 | 1 PE, 66 DVT | 22% |
| Poyiadji, N. (32) | 328 | 61 | 54**%** | 47% ≥30 kg/m^2^ | hypertension (60%), DM (39%), smoking (37%), cancer (14%), VTE history (8%) | - | 22.0 | - | 72 PE | 9% |
| Criel, M. (33) | 82 | 64 | 41**%** | 30 | hypertension (37%), DM (17%) | - | - | 7.3 | 6 DVT | 11% |
| Poissy, J. (34) | 107 | 57* | 38**%** | 30* | n.r. | 22.4 | 20.6 | 4.7 | 19 PE, 3 PE+DVT, 2 DVT | n.r. |
| Lodigiani, C. (35) | 362 | 66 | 32**%** | 22% ≥30 kg/m^2^ | hypertension (47%), DM (23%), CKD (16%), CAD (14%), smoking (11%), active cancer (6%), | 4.4 | 2.8 | 1.7 | 10 PE, 6 DVT | 25% |
| Grillet, F. (36) | 280 | 66 | 30**%** | n.r. | CVD (39%), DM (20%), cancer (20%), chronic respiratory disease (15%) | - | 8.2 | - | 23 PE | n.r. |
| Leonard-Lorant, I. (37) | 106 | 63 | 34**%** | 28 | n.r. | - | 30.2 | - | 32 PE | n.r. |
| Longchamp, A. (38) | 25 | 68 | 36**%** | 27.5 | hypertension (40%), smoking (24%), CVD (12%), OSAS (12%), DM (4%), COPD (8%),), active cancer (8%) | 32.0 | 20.0 | 24.0 | 2 PE, 3 PE+DVT, 3 DVT | 20% |
| Goyal, P. (39) | 393 | 62 | 39**%** | 35% ≥30 kg/m^2^ | hypertension (50%), DM (25%), CAD (14%), Asthma (13%), COPD (5%) | 3.3 | - | - | - | 10% |
| Beigel, J. (40) | 1063 | 59 | 36**%** | 32% obese | hypertension (50%), DM (31%), CAD (12%), asthma (11%), cancer (8%), CKD (6%), CHF (5%) | 2.0 | 0.6 | 1.4 | 6 PE, 15 DVT | 8% |
| Tremblay, D. (41) | 3772 | 57 | 45**%** | 4.1% obese | lung disease (15%), smoking (4%), prior VTE (3%) | 1.2 | - | - | - | 15% |
| Archer, J.E. (42) | 1115 | 31% between 50-69 | 46**%** | n.r. | hypertension (50%), DM (25%), cancer (17%), CKD (15%), COPD (10%), smoking (9%), CHF (8%), cerebrovascular disease (8%) | - | 2.0 | - | 22 PE | n.r. |
| Huet, T. (43) | 96 | 71 | 36**%** | 25.5 in treatment, 29.0 in control | hypertension (60%), DM (30%), cardiopathy (20%), pulmonary disease (20%) | 15.6 | - | - | - | n.r. |
| Bowles, L. (44) | 35 | 57 | 31**%** | n.r. | n.r. | 5.7 | 2.9 | 2.9 | 1 PE, 1 DVT | 23% |
| Nahum, J. (45) | 34 | 62 | 26**%** | 31.4 | DM (44%), hypertension (38%), ischemic heart disease (9%), COPD (6%), cancer (3%), | - | - | 79.4 | 27 DVT | n.r. |
| Zerwes, S. (46) | 20 | 64 | 30**%** | 28.1 | hypertension (65%), CAD (30%), hyperlipidemia (20%), prior VTE (15%), DM (10%), smoking (10%), | 20.0 | 5.0 | 20.0 | 0 PE, 1 PE+DVT, 3 DVT | 25% |
| Betoule, A. (47) | 76 | 62 | n.r. | n.r. | hypertension (33%), DM (13%) | - | 2.6 | - | 2 PE | 4% |
| Gervaise, A. (48) | 72 | 62 | 25**%** | 26.7 | n.r. | - | 18.1 | - | 13 PE | 15% |
| Grandmaison, G. (49) | 58 | 65 | 19**%** | 28 | cancer (3%), prior VTE (3%) | 39.7 | 6.9 | - | 4 PE | n.r. |
| Le Jeune, S. (50) | 42 | 65 | 45**%** | 28 | hypertension (48%), DM (31%), CVD (17%), smoking (12%), active cancer (7%) | 31.0 | 9.5 | 19.0 | 3 PE, 1 PE+DVT, 7 DVT | 4.8% |
| Taccone, F. (51) | 82 | n.r. | n.r. | n.r. |  | - | 15.9 | - | 13 PE | n.r. |
| Violi, F. (52) | 73 | 67 | 19% | n.r. | n.r. | - | 2.7 | - | 2 PE | n.r. |
| Blumfield, E. (53) | 16 | 9.2 | 38% | 25% obese | Asthma (19%) | - | 12.5 | - | 2 PE | 0% |
| Santoliquido, A. (54) | 84 | 68 | 27% | 18% obese | hypertension (54%), DM (21%), cancer (17%), CAD/CVD (13%), prior VTE (4%) | - | - | 11.9 | 10 DVT | 10% |
| Mestre-Gómez, B. (55) | 452 | n.r. | n.r. | n.r. |  | - | 6.4 | - | 29 PE | n.r. |
| Patell, R. (56) | 398 | no cancer 61, cancer 69 | 47% | n.r. | hypertension (55%), DM (35%), heart disease (26%), active cancer (11%) | - | 2.5 | 3.8 | 10 PE, 15 DVT | 21% |
| Berger, J. (57) | 2377 | 64 | 39% | n.r. | hypertension (62%), DM (39%), CAD (22%), heart failure (13%), cancer (10%) | - | 2.9 | 4.3 | 68 PE, 103 DVT | 26% |
| Dubois-Silva, Á. (58) | 171 | n.r. | n.r. | n.r. |  | - | 4.7 | - | 8 PE | n.r. |
| Fauvel, C. (59) | 2878 | 67 | 42% | 27.3 | hypertension (51%), DM (24%), cancer (14%), CAD (13%), CHF (11%) | 4.9 | 3.6 | 1.3 | 103 PE, 37 DVT | 13% |
| Whyte, M. (60) | 1477 | n.r. | n.r. |  |  | - | 5.4 | - | 80 PE | n.r. |
| Rieder, M. (61) | 49 | 60 | 39% | 26.6 | cancer (22%), prior VTE (12%) | 6.1 | 4.1 | - | 2 PE | 7% |
| Mattioli, M. (62) | 105 | 74 | 42% | 14% obese | hypertension (55%), DM (12%), smoking (10%) | 1.0 | 1.0 | 0.0 | 1 PE | 21% |
| Pesavento, R. (63) | 324 | 71 | 44% | 15% obese | Prior VTE (5%) | 2.8 | 0.3 | 2.5 | 1 PE, 8 DVT | 13% |
| Bilaloglu, S. (64) | 3334 | 64 | 40% | n.r. | hypertension (50%), DM (37%), CAD (19%), CHF (8%) | 7.0 | 3.2 | 3.9 | 106 PE, 129 DVT | 25% |
| Hanif, A. (65) | 921 | 62 | 38% | 30.4 | n.r. | 1.7 | - | - | n.r. | 35% |
| Cho, E. (66) | 158 | 67 | 46% | 29.5 | Hypertension (72%), DM (43%), smoking (11%), CHF (7%) | - | - | 32.9 | 52 DVT | n.r. |
| Zermatten, M. (67) | 100 | 64 | 26% | 26% obese | hypertension (53%), DM (26%), cardiopathy (24%), cancer (3%) | 22.0 | 15.0 | 7.0 | 15 PE, 7 DVT | 28% |
| Moll, M. (68) | 210 | 62 | 52% | 29.8 | hypertension (60%), DM (33%), asthma (17%), CAD (9%), CHF (9%) | 4.3 | 3.3 | 1.0 | 7 PE, 2 DVT | 17% |
| Chen, J. (69) | 1008 | 65 | 40% | n.r. | Hypertension (10%), smoking (6%), DM (5%), CVD (4%) | 1.0 | - | - | 10 PE | 24% |
| Trimaille, A. (70) | 289 | 62 | 20% | n.r. | n.r. | 17.0 | 14.5 | 4.2 | 42 PE, 12 DVT | 8% |
| Naymagon, L. (71) | 1065 | 66 | 40% | 37% BMI > 30 | prior VTE (5%), smoking (4%) | 2.8 | - | - | n.r. | n.r. |
| Freund, Y. (72) | 974 | n.r | n.r. | n.r | n.r | - | 15.2 | - | 148 PE | n.r. |
| Lendorf, M. (73) | 111 | 69 | 40% | 26 | Smoking (39%), hypertension (34%), DM (15%), cerebrovascular disease (12%), asthma (11%), immunosuppression (9%), cancer (8%) | - | 8.1 | - | 9 PE | 14% |
| Marone, E. (74) | 101 | 65 | n.r. | n.r. | n.r. | - | - | 41.6 | 42 DVT | n.r. |
| Lachant, D. (75) | 107 | 78 | 55% | 29 | AF (71%), smoking (59%), CHF (32%), DM (30%)CAD (28%), prior VTE (25%), CKD (25%), COPD (14%) | 0.0 | 0.0 | 0.0 | 0 PE, 0 DVT | 12% |
| Koleilat, I. (76) | 3404 | 64 | 47% | n.r. | hypertension (70%), hyperlipidemia (37%), DM (37%), smoking (31%), CKD (21%), CAD (12%) | - | - | 0.5 | 18 DVT | n.r. |
| Fox, T. (77) | 55 | 63 | 31% | 25.7 | Cancer (95%), hypertension (37%), DM (20%), smoking (19%), CKD (13%) | 10.9 | 5.5 | 5.5 | 3 PE, 3 DVT | 35% |
| Mei, F. (78) | 256 | 56 | 49% | n.r. | hypertension (23%), DM (18%), acute liver dysfunction (5%), cancer (2%) | 2.0 | 0.4 | 1.6 | 1 PE, 4 DVT | 6.3% |
| Aleva, F.E. (79) | 50 | 65 | 34% | n.r. | hypertension (34%), smoking (30%), DM (16%), CAD (10%) | 36.0 | 26.0 | 2.0 | 13 PE, 1 DVT, 5 CRT | 26% |
| Fredi, M. (80) | 88 | 70 | 51% | n.r. | hypertension (60%), CVD (24%), DM (16%), cancer (10%), chronic lung disease (10%), CKD (7%) | 5.7 | 4.5 | 1.1 | 4 PE, 1 DVT | 11% |
| Pizzolo, F. (81) | 43 | 66 | 33% | n.r. | hypertension (53%), CVD (19%), hyperlipidemia (19%), DM (14%), active cancer (9%) | - | - | 27.9 | 9 DVT | n.r. |
| Larsen, K. (82) | 35 | 66 | 23% | n.r. | CHF (26%), COPD (17%), prior VTE (9%) | 20.0 | 14.3 | 5.7 | 5 PE, 2 DVT | n.r. |
| Chen, S. (83) | 88 | 63 | 39% | n.r. | hypertension (35%), DM (10%), cancer (6%), cerebrovascular disease (3%) | - | - | 45.5 | 40 DVT | n.r. |
| Ierardi, A. (84) | 234 | 61 | 70% | 29.08 | Smoking (87%), hypertension (40%), DM (17%), COPD (8%), ischemic heart disease (7%) | 10.7 | - | 10.7 | 25 DVT | n.r. |
| Soumange, T. (85) | 375 | 64 | 23% | 40% BMI > 30 | hypertension (58%), DM (26%), cancer (12%), CAD (10%) | 21.1 | 14.7 | 9.3 | 55 PE, 24 DVT | 36% (ICU) |

Abb.: AF, atrial fibrillation; BMI, body mass index; CAD, coronary artery disease; CHF, congestive heart failure; CKD, chronic kidney disease; COPD, chronic obstructive pulmonary disease; CRT, catheter related thrombosis; CVD, cardiovascular disease; DM, diabetes mellitus type 2; DVT, deep vein thrombosis; n.r., not reported; OSAS, obstructive sleep apnoea syndrome; PE, pulmonary embolism; VTE, venous thromboembolism.

*only reported for VTE patients

## Table S3: Quality of identified studies

| **First Author** | Criteria | | | | | | | | | Overall appraisal |
| --- | --- | --- | --- | --- | --- | --- | --- | --- | --- | --- |
|  | C1 | C2 | C3 | C4 | C5 | C6 | C7 | C8 | C9 |  |
| Maatman, T.K. | Y | Y | Y | Y | Y | Y | Y | U | Y | Include |
| Desborough, M.J.R. | Y | Y | Y | Y | Y | Y | Y | U | Y | Include |
| Galeano-Valle, F. | Y | Y | Y | Y | Y | Y | Y | Y | Y | Include |
| Inciardi, R.M. | U | Y | Y | Y | Y | U | U | U | Y | Include |
| Artifoni, M. | N | Y | Y | Y | Y | Y | Y | U | Y | Exclude |
| Wang, Y. | U | Y | Y | Y | Y | U | U | U | Y | Include |
| Ren, B. | Y | Y | U | Y | Y | Y | Y | U | Y | Include |
| Demelo-Rodríguez, P. | N | Y | Y | Y | Y | Y | Y | Y | Y | Exclude |
| Helms, J. | Y | Y | Y | Y | Y | Y | Y | U | Y | Include |
| Llitjos, J.-F. | Y | Y | N | Y | Y | Y | Y | U | Y | Include |
| Stoneham, S.M. | Y | Y | Y | U | Y | Y | Y | U | Y | Include |
| Wright, F.L. | Y | Y | U | Y | Y | Y | Y | U | Y | Include |
| Louhaichi, S. | Y | Y | N | N | Y | U | U | U | U | Exclude |
| Thomas, W. | Y | Y | Y | Y | Y | Y | Y | Y | Y | Include |
| Bombard, F. | N | Y | Y | N | Y | Y | Y | Y | Y | Exclude |
| Faggiano, P. | Y | Y | N | Y | Y | Y | Y | U | Y | Include |
| Klok, F.A. | Y | Y | Y | Y | Y | Y | Y | Y | Y | Include |
| Middeldorp, S. | Y | Y | Y | Y | Y | Y | Y | Y | Y | Include |
| Tavazzi, G. | Y | Y | U | N | Y | Y | Y | U | Y | Include |
| Beun, R. | Y | Y | Y | N | Y | Y | Y | U | Y | Include |
| Cui, S. | Y | Y | Y | Y | Y | Y | Y | U | Y | Include |
| Spieza, L. | Y | Y | N | N | Y | U | U | U | Y | Include |
| Al-Samkari, H. | Y | Y | Y | Y | Y | Y | Y | Y | Y | Include |
| Fraissé, M. | Y | Y | Y | Y | Y | U | U | U | Y | Include |
| Hippensteel, J. | U | Y | Y | Y | Y | Y | Y | U | Y | Include |
| Voicu, S. | Y | Y | U | Y | Y | Y | Y | U | Y | Include |
| Guo, T. | U | Y | Y | Y | Y | U | U | U | Y | Include |
| Campochiaro | U | U | Y | Y | Y | Y | Y | U | Y | Include |
| Zhang, L. | Y | Y | Y | Y | Y | Y | Y | U | Y | Include |
| Poyiadji, N. | N | Y | Y | Y | Y | Y | Y | Y | Y | Exclude |
| Criel, M. | Y | Y | Y | N | Y | U | N | U | Y | Exclude |
| Poissy, J. | Y | Y | Y | N | Y | Y | Y | Y | Y | Include |
| Lodigiani, C. | Y | Y | Y | Y | Y | Y | Y | Y | Y | Include |
| Grillet, F. | Y | Y | Y | U | Y | Y | Y | Y | Y | Include |
| Leonard-Lorant, I. | N | Y | Y | N | Y | Y | Y | Y | Y | Exclude |
| Longchamp, A. | Y | Y | N | Y | Y | Y | Y | U | Y | Include |
| Goyal, P. | Y | Y | Y | Y | Y | U | U | U | Y | Include |
| Beigel, J. | U | U | Y | Y | Y | U | U | U | Y | Include |
| Tremblay, D. | Y | Y | Y | Y | Y | U | U | U | Y | Include |
| Archer, J.E. | N | N | Y | Y | Y | U | U | U | Y | Exclude |
| Huet, T. | U | U | Y | Y | Y | U | U | U | Y | Include |
| Bowles, L. | N | Y | Y | U | Y | Y | Y | U | Y | Exclude |
| Nahum, J. | Y | Y | Y | Y | Y | Y | Y | U | Y | Include |
| Zerwes, S. | Y | Y | N | Y | Y | Y | Y | U | Y | Include |
| Betoule, A. | Y | Y | Y | N | Y | U | U | U | Y | Include |
| Gervaise, A. | N | Y | Y | Y | Y | Y | Y | U | Y | Exclude |
| Grandmaison, G. | Y | Y | U | N | Y | Y | Y | U | Y | Include |
| Le Jeune, S. | Y | Y | U | Y | Y | Y | Y | U | Y | Include |
| Taccone, F. | Y | Y | Y | Y | Y | Y | Y | U | y | Include |
| Violi, F. | Y | Y | Y | U | Y | U | U | U | Y | Include |
| Blumfield, E. | N | Y | N | Y | Y | U | U | U | Y | Exclude |
| Santoliquido, A. | Y | Y | Y | Y | Y | Y | Y | U | Y | Include |
| Mestre-Gómez, B. | Y | Y | Y | Y | Y | Y | Y | Y | Y | Include |
| Patell, R. | Y | Y | Y | Y | Y | Y | Y | Y | Y | Include |
| Berger, J. | Y | Y | Y | Y | Y | Y | Y | Y | Y | Include |
| Dubois-Silva, Á. | Y | Y | Y | U | Y | Y | Y | Y | Y | Include |
| Fauvel, C. | Y | Y | Y | Y | Y | Y | Y | Y | Y | Include |
| Whyte, M. | Y | Y | Y | Y | Y | Y | Y | Y | Y | Include |
| Rieder, M. | Y | Y | U | Y | Y | Y | Y | U | Y | Include |
| Mattioli, M. | Y | Y | Y | Y | Y | Y | Y | U | Y | Include |
| Pesavento, R. | Y | Y | Y | Y | Y | Y | Y | Y | Y | Include |
| Bilaloglu, S. | Y | Y | Y | Y | Y | Y | Y | Y | Y | Include |
| Hanif, A. | Y | Y | Y | U | Y | Y | Y | U | Y | Include |
| Cho, E. | N | Y | Y | Y | Y | Y | Y | U | Y | Exclude |
| Zermatten, M. | Y | Y | Y | U | Y | Y | Y | U | Y | Include |
| Moll, M. | Y | Y | Y | Y | Y | Y | Y | U | Y | Include |
| Chen, J. | Y | Y | Y | U | N | U | U | U | Y | Exclude |
| Trimaille, A. | y | y | y | u | y | y | y | u | y | Include |
| Naymagon, L. | N | Y | Y | Y | Y | Y | Y | U | Y | Exclude |
| Freund, Y. | N | Y | Y | N | Y | Y | Y | Y | Y | Exclude |
| Lendorf, M. | Y | Y | Y | Y | Y | Y | Y | U | Y | Include |
| Marone, E. | N | Y | Y | N | Y | Y | Y | U | Y | Exclude |
| Lachant, D. | N | Y | Y | U | Y | Y | Y | U | Y | Exclude |
| Koleilat, I. | Y | Y | Y | N | Y | Y | Y | U | Y | Include |
| Fox, T. | N | U | U | Y | Y | U | U | U | Y | Exclude |
| Mei, F. | Y | Y | Y | U | Y | Y | Y | U | Y | Include |
| Aleva, F.E. | Y | Y | U | Y | Y | U | U | U | Y | Include |
| Fredi, M. | Y | Y | Y | U | Y | U | U | U | Y | Include |
| Pizzolo, F. | Y | Y | U | Y | Y | Y | Y | Y | Y | Include |
| Larsen, K. | Y | Y | U | Y | Y | U | U | U | Y | Include |
| Chen, S. | N | U | Y | Y | Y | Y | Y | U | Y | Exclude |
| Ierardi, A. | Y | Y | Y | Y | Y | Y | Y | U | Y | Include |
| Soumange, T. | Y | Y | Y | Y | Y | U | U | U | Y | Include |
| Gatto, M. | U | U | Y | Y | Y | U | U | U | Y | Include |
| Mazzaccaro, D. | U | U | U | Y | Y | Y | Y | U | Y | Include |
| Cattaneo, M. | Y | Y | Y | N | Y | U | U | U | Y | Include |

# References

1. Munn Z, Moola S, Lisy K, Riitano D, Tufanaru C. Methodological guidance for systematic reviews of observational epidemiological studies reporting prevalence and cumulative incidence data. Int J Evid Based Healthc. 2015;13(3):147-53.

2. Maatman TK, Jalali F, Feizpour C, Douglas A, 2nd, McGuire SP, Kinnaman G, et al. Routine Venous Thromboembolism Prophylaxis May Be Inadequate in the Hypercoagulable State of Severe Coronavirus Disease 2019. Critical care medicine. 2020:10.1097/CCM.0000000000004466.

3. Desborough MJR, Doyle AJ, Griffiths A, Retter A, Breen KA, Hunt BJ. Image-proven thromboembolism in patients with severe COVID-19 in a tertiary critical care unit in the United Kingdom. Thrombosis research. 2020;193:1-4.

4. Galeano-Valle F, Oblitas CM, Ferreiro-Mazón MM, Alonso-Muñoz J, Del Toro-Cervera J, di Natale M, et al. Antiphospholipid antibodies are not elevated in patients with severe COVID-19 pneumonia and venous thromboembolism. Thrombosis research. 2020;192:113-5.

5. Inciardi RM, Adamo M, Lupi L, Cani DS, Di Pasquale M, Tomasoni D, et al. Characteristics and outcomes of patients hospitalized for COVID-19 and cardiac disease in Northern Italy. European Heart Journal. 2020;41(19):1821-9.

6. Artifoni M, Danic G, Gautier G, Gicquel P, Boutoille D, Raffi F, et al. Systematic assessment of venous thromboembolism in COVID-19 patients receiving thromboprophylaxis: incidence and role of D-dimer as predictive factors. Journal of thrombosis and thrombolysis. 2020;50(1):211-6.

7. Wang Y, Zhang D, Du G, Du R, Zhao J, Jin Y, et al. Remdesivir in adults with severe COVID-19: a randomised, double-blind, placebo-controlled, multicentre trial. The Lancet. 2020;395(10236):1569-78.

8. Ren B, Yan F, Deng Z, Zhang S, Xiao L, Wu M, et al. Extremely High Incidence of Lower Extremity Deep Venous Thrombosis in 48 Patients with Severe COVID-19 in Wuhan. Circulation.0(0).

9. Demelo-Rodríguez P, Cervilla-Muñoz E, Ordieres-Ortega L, Parra-Virto A, Toledano-Macías M, Toledo-Samaniego N, et al. Incidence of asymptomatic deep vein thrombosis in patients with COVID-19 pneumonia and elevated D-dimer levels. Thrombosis research. 2020;192:23-6.

10. Helms J, Tacquard C, Severac F, Leonard-Lorant I, Ohana M, Delabranche X, et al. High risk of thrombosis in patients with severe SARS-CoV-2 infection: a multicenter prospective cohort study. Intensive Care Medicine. 2020;46(6):1089-98.

11. Llitjos J-F, Leclerc M, Chochois C, Monsallier J-M, Ramakers M, Auvray M, et al. High incidence of venous thromboembolic events in anticoagulated severe COVID-19 patients. Journal of Thrombosis and Haemostasis.n/a(n/a).

12. Stoneham SM, Milne KM, Nuttal E, Frew GH, Sturrock BR, Sivaloganathan H, et al. Thrombotic risk in COVID-19: a case series and case–control study. Clinical Medicine. 2020:clinmed.2020-0228.

13. Wright FL, Vogler TO, Moore EE, Moore HB, Wohlauer MV, Urban S, et al. Fibrinolysis Shutdown Correlation with Thromboembolic Events in Severe COVID-19 Infection. Journal of the American College of Surgeons.

14. Louhaichi S, Allouche A, Baili H, Jrad S, Radhouani A, Greb D, et al. Features of patients with 2019 novel coronavirus admitted in a pneumology department: The first retrospective Tunisian case series. Tunis Med. 2020;98(4):261-5.

15. Thomas W, Varley J, Johnston A, Symington E, Robinson M, Sheares K, et al. Thrombotic complications of patients admitted to intensive care with COVID-19 at a teaching hospital in the United Kingdom. Thrombosis research. 2020;191:76-7.

16. Bompard F, Monnier H, Saab I, Tordjman M, Abdoul H, Fournier L, et al. Pulmonary embolism in patients with Covid-19 pneumonia. European Respiratory Journal. 2020:2001365.

17. Faggiano P, Bonelli A, Paris S, Milesi G, Bisegna S, Bernardi N, et al. Acute pulmonary embolism in COVID-19 disease: Preliminary report on seven patients. International Journal of Cardiology. 2020;313:129-31.

18. Klok FA, Kruip MJHA, van der Meer NJM, Arbous MS, Gommers DAMPJ, Kant KM, et al. Incidence of thrombotic complications in critically ill ICU patients with COVID-19. Thrombosis research. 2020;191:145-7.

19. Klok FA, Kruip MJHA, van der Meer NJM, Arbous MS, Gommers D, Kant KM, et al. Confirmation of the high cumulative incidence of thrombotic complications in critically ill ICU patients with COVID-19: An updated analysis. Thrombosis research. 2020;191:148-50.

20. Middeldorp S, Coppens M, van Haaps TF, Foppen M, Vlaar AP, Müller MCA, et al. Incidence of venous thromboembolism in hospitalized patients with COVID-19. Journal of Thrombosis and Haemostasis.n/a(n/a).

21. Tavazzi G, Civardi L, Caneva L, Mongodi S, Mojoli F. Thrombotic events in SARS-CoV-2 patients: an urgent call for ultrasound screening. Intensive Care Medicine. 2020;46(6):1121-3.

22. Beun R, Kusadasi N, Sikma M, Westerink J, Huisman A. Thromboembolic events and apparent heparin resistance in patients infected with SARS-CoV-2. International Journal of Laboratory Hematology. 2020;42(S1):19-20.

23. Cui S, Chen S, Li X, Liu S, Wang F. Prevalence of venous thromboembolism in patients with severe novel coronavirus pneumonia. Journal of Thrombosis and Haemostasis. 2020;18(6):1421-4.

24. Spiezia L, Boscolo A, Poletto F, Cerruti L, Tiberio I, Campello E, et al. COVID-19-Related Severe Hypercoagulability in Patients Admitted to Intensive Care Unit for Acute Respiratory Failure. Thrombosis and haemostasis. 2020;120(6):998-1000.

25. Al-Samkari H, Karp Leaf RS, Dzik WH, Carlson JC, Fogerty AE, Waheed A, et al. COVID and Coagulation: Bleeding and Thrombotic Manifestations of SARS-CoV2 Infection. Blood. 2020.

26. Fraissé M, Logre E, Pajot O, Mentec H, Plantefève G, Contou D. Thrombotic and hemorrhagic events in critically ill COVID-19 patients: a French monocenter retrospective study. Critical Care. 2020;24(1):275.

27. Hippensteel JA, Burnham EL, Jolley SE. Prevalence of venous thromboembolism in critically ill patients with COVID-19. British Journal of Haematology.n/a(n/a).

28. Voicu S, Bonnin P, Stépanian A, Chousterman BG, Le Gall A, Malissin I, et al. High prevalence of deep vein thrombosis in mechanically ventilated COVID-19 patients. Journal of the American College of Cardiology. 2020:27399.

29. Guo T, Shen Q, Guo W, He W, Li J, Zhang Y, et al. Clinical Characteristics of Elderly Patients with COVID-19 in Hunan Province, China: A Multicenter, Retrospective Study. Gerontology. 2020.

30. Campochiaro C, Della-Torre E, Cavalli G, De Luca G, Ripa M, Boffini N, et al. Efficacy and safety of tocilizumab in severe COVID-19 patients: a single-centre retrospective cohort study. European journal of internal medicine. 2020;76:43-9.

31. Zhang L, Feng X, Zhang D, Jiang C, Mei H, Wang J, et al. Deep Vein Thrombosis in Hospitalized Patients with Coronavirus Disease 2019 (COVID-19) in Wuhan, China: Prevalence, Risk Factors, and Outcome. Circulation.0(0).

32. Poyiadji N, Cormier P, Patel PY, Hadied MO, Bhargava P, Khanna K, et al. Acute Pulmonary Embolism and COVID-19. Radiology.0(0):201955.

33. Criel M, falter M, Jaeken J, Van Kerrebroeck M, Lefere I, Meylaerts L, et al. Venous thromboembolism in SARS-CoV-2 patients: only a problem in ventilated ICU patients, or is there more to it? European Respiratory Journal. 2020:2001201.

34. Poissy J, Goutay J, Caplan M, Parmentier E, Duburcq T, Lassalle F, et al. Pulmonary Embolism in COVID-19 Patients: Awareness of an Increased Prevalence. Circulation.0(0).

35. Lodigiani C, Iapichino G, Carenzo L, Cecconi M, Ferrazzi P, Sebastian T, et al. Venous and arterial thromboembolic complications in COVID-19 patients admitted to an academic hospital in Milan, Italy. Thrombosis research. 2020;191:9-14.

36. Grillet F, Behr J, Calame P, Aubry S, Delabrousse E. Acute Pulmonary Embolism Associated with COVID-19 Pneumonia Detected by Pulmonary CT Angiography. Radiology.0(0):201544.

37. Leonard-Lorant I, Delabranche X, Severac F, Helms J, Pauzet C, Collange O, et al. Acute Pulmonary Embolism in COVID-19 Patients on CT Angiography and Relationship to D-Dimer Levels. Radiology.0(0):201561.

38. Longchamp A, Longchamp J, Manzocchi-Besson S, Whiting L, Haller C, Jeanneret S, et al. Venous Thromboembolism in Critically Ill Patients with Covid-19: Results of a Screening Study for Deep Vein Thrombosis. Research and Practice in Thrombosis and Haemostasis.n/a(n/a).

39. Goyal P, Choi JJ, Pinheiro LC, Schenck EJ, Chen R, Jabri A, et al. Clinical Characteristics of Covid-19 in New York City. New England Journal of Medicine. 2020;382(24):2372-4.

40. Beigel JH, Tomashek KM, Dodd LE, Mehta AK, Zingman BS, Kalil AC, et al. Remdesivir for the Treatment of Covid-19 — Preliminary Report. New England Journal of Medicine. 2020.

41. Tremblay D, van Gerwen M, Alsen M, Thibaud S, Kessler AJ, Venugopal S, et al. Impact of anticoagulation prior to COVID-19 infection: a propensity score-matched cohort study. Blood. 2020.

42. Archer JE, Odeh A, Ereidge S, Salem HK, Jones GP, Gardner A, et al. Mortality and pulmonary complications in patients undergoing surgery with perioperative SARS-CoV-2 infection: an international cohort study. The Lancet.

43. Huet T, Beaussier H, Voisin O, Jouveshomme S, Dauriat G, Lazareth I, et al. Anakinra for severe forms of COVID-19: a cohort study. The Lancet Rheumatology. 2020;2(7):e393-e400.

44. Bowles L, Platton S, Yartey N, Dave M, Lee K, Hart DP, et al. Lupus Anticoagulant and Abnormal Coagulation Tests in Patients with Covid-19. New England Journal of Medicine. 2020.

45. Nahum J, Morichau-Beauchant T, Daviaud F, Echegut P, Fichet J, Maillet J-M, et al. Venous Thrombosis Among Critically Ill Patients With Coronavirus Disease 2019 (COVID-19). JAMA Network Open. 2020;3(5):e2010478-e.

46. Zerwes S, Hernandez Cancino F, Liebetrau D, Gosslau Y, Warm T, Märkl B, et al. [Increased risk of deep vein thrombosis in intensive care unit patients with CoViD-19 infections?-Preliminary data]. Chirurg. 2020:1-7.

47. Betoule A, Martinet C, Gasperini G, Muller P, Foucher S, Benner P, et al. Diagnosis of venous and arterial thromboembolic events in COVID-19 virus-infected patients. Journal of thrombosis and thrombolysis. 2020:1-3.

48. Gervaise A, Bouzad C, Peroux E, Helissey C. Acute pulmonary embolism in non-hospitalized COVID-19 patients referred to CTPA by emergency department. Eur Radiol. 2020:1-8.

49. Grandmaison G, Andrey A, Périard D, Engelberger RP, Carrel G, Doll S, et al. Systematic Screening for Venous Thromboembolic Events in COVID-19 Pneumonia. TH open : companion journal to thrombosis and haemostasis. 2020;4(2):e113-e5.

50. Le Jeune S, Suhl J, Benainous R, Minvielle F, Purser C, Foudi F, et al. High prevalence of early asymptomatic venous thromboembolism in anticoagulated COVID-19 patients hospitalized in general wards. Journal of thrombosis and thrombolysis. 2020:1-5.

51. Taccone FS, Gevenois PA, Peluso L, Pletchette Z, Lheureux O, Brasseur A, et al. Higher Intensity Thromboprophylaxis Regimens and Pulmonary Embolism in Critically Ill Coronavirus Disease 2019 Patients. Critical care medicine. 2020:10.1097/CCM.0000000000004548.

52. Violi F, Ceccarelli G, Cangemi R, Alessandri F, D’Ettorre G, Oliva A, et al. Hypoalbuminemia, Coagulopathy, and Vascular Disease in COVID-19. Circulation Research. 2020;127(3):400-1.

53. Blumfield E, Levin TL, Kurian J, Lee EY, Liszewski MC. Imaging Findings in Multisystem Inflammatory Syndrome in Children (MIS-C) Associated with COVID-19. American Journal of Roentgenology. 2020.

54. Santoliquido A, Porfidia A, Nesci A, De Matteis G, Marrone G, Porceddu E, et al. Incidence of deep vein thrombosis among non-ICU patients hospitalized for COVID-19 despite pharmacological thromboprophylaxis. Journal of Thrombosis and Haemostasis.n/a(n/a).

55. Mestre-Gómez B, Lorente-Ramos RM, Rogado J, Franco-Moreno A, Obispo B, Salazar-Chiriboga D, et al. Incidence of pulmonary embolism in non-critically ill COVID-19 patients. Predicting factors for a challenging diagnosis. Journal of thrombosis and thrombolysis. 2020:1-7.

56. Patell R, Bogue T, Bindal P, Koshy A, Merrill M, Aird WC, et al. Incidence of thrombosis and hemorrhage in hospitalized cancer patients with COVID-19. Journal of Thrombosis and Haemostasis.n/a(n/a).

57. Berger JS, Kunichoff D, Adhikari S, Ahuja T, Amoroso N, Aphinyaphongs Y, et al. Prevalence and Outcomes of D-Dimer Elevation in Hospitalized Patients With COVID-19. Arteriosclerosis, Thrombosis, and Vascular Biology.0(0):ATVBAHA.120.314872.

58. Dubois-Silva Á, Barbagelata-López C, Mena Á, Piñeiro-Parga P, Llinares-García D, Freire-Castro S. Pulmonary embolism and screening for concomitant proximal deep vein thrombosis in noncritically ill hospitalized patients with coronavirus disease 2019. Internal and Emergency Medicine. 2020;15(5):865-70.

59. Fauvel C, Weizman O, Trimaille A, Mika D, Pommier T, Pace N, et al. Pulmonary embolism in COVID-19 patients: a French multicentre cohort study. European Heart Journal. 2020.

60. Whyte MB, Kelly PA, Gonzalez E, Arya R, Roberts LN. Pulmonary embolism in hospitalised patients with COVID-19. Thrombosis research. 2020;195:95-9.

61. Rieder M, Goller I, Jeserich M, Baldus N, Pollmeier L, Wirth L, et al. Rate of venous thromboembolism in a prospective all-comers cohort with COVID-19. Journal of Thrombosis and Thrombolysis. 2020.

62. Mattioli M, Benfaremo D, Mancini M, Mucci L, Mainquà P, Polenta A, et al. Safety of intermediate dose of low molecular weight heparin in COVID-19 patients. Journal of Thrombosis and Thrombolysis. 2020.

63. Pesavento R, Ceccato D, Pasquetto G, Monticelli J, Leone L, Frigo A, et al. The hazard of (sub)therapeutic doses of anticoagulants in non-critically ill patients with Covid-19: The Padua province experience. Journal of Thrombosis and Haemostasis.n/a(n/a).

64. Bilaloglu S, Aphinyanaphongs Y, Jones S, Iturrate E, Hochman J, Berger JS. Thrombosis in Hospitalized Patients With COVID-19 in a New York City Health System. JAMA. 2020;324(8):799-801.

65. Hanif A, Khan S, Mantri N, Hanif S, Saleh M, Alla Y, et al. Thrombotic complications and anticoagulation in COVID-19 pneumonia: a New York City hospital experience. Ann Hematol. 2020.

66. Cho ES, McClelland PH, Cheng O, Kim Y, Hu J, Zenilman ME, et al. Utility of d-dimer for diagnosis of deep vein thrombosis in coronavirus disease-19 infection. Journal of Vascular Surgery: Venous and Lymphatic Disorders. 2020.

67. Zermatten MG, Pantet O, Gomez F, Schneider A, Méan M, Mazzolai L, et al. Utility of D-dimers and intermediate-dose prophylaxis for venous thromboembolism in critically ill patients with COVID-19. Thrombosis research.

68. Moll M, Zon RL, Sylvester KW, Chen EC, Cheng V, Connell NT, et al. VTE in ICU Patients With COVID-19. Chest. 2020.

69. Chen J, Wang X, Zhang S, Lin B, Wu X, Wang Y, et al. Characteristics of Acute Pulmonary Embolism in Patients With COVID-19 Associated Pneumonia From the City of Wuhan. Clinical and Applied Thrombosis/Hemostasis. 2020;26:1076029620936772.

70. Trimaille A, Curtiaud A, Marchandot B, Matsushita K, Sato C, Leonard-Lorant I, et al. Venous thromboembolism in non-critically ill patients with COVID-19 infection. Thrombosis research. 2020;193:166-9.

71. Naymagon L, Zubizarreta N, Feld J, van Gerwen M, Alsen M, Thibaud S, et al. Admission D-dimer levels, D-dimer trends, and outcomes in COVID-19. Thrombosis research. 2020;196:99-105.

72. Freund Y, Drogrey M, Miró Ò, Marra A, Féral-Pierssens A-L, Penaloza A, et al. Association between Pulmonary Embolism and COVID-19 in ED patients Undergoing CTPA: the PEPCOV international retrospective study. Academic Emergency Medicine.n/a(n/a).

73. Lendorf ME, Boisen MK, Kristensen PL, Løkkegaard ECL, Krog SM, Brandi L, et al. Characteristics and early outcomes of patients hospitalised for COVID-19 in North Zealand, Denmark. Dan Med J. 2020;67(9).

74. Marone EM, Bonalumi G, Curci R, Arzini A, Chierico S, Marazzi G, et al. Characteristics of Venous Thromboembolism in COVID-19 Patients: A Multicenter Experience from Northern Italy. Annals of Vascular Surgery. 2020.

75. Lachant DJ, Lachant NA, Kouides P, Rappaport S, Prasad P, White RJ. Chronic therapeutic anticoagulation is associated with decreased thrombotic complications in SARS-CoV-2 infection. Journal of Thrombosis and Haemostasis.n/a(n/a).

76. Koleilat I, Galen B, Choinski K, Hatch AN, Jones DB, Billett H, et al. Clinical characteristics of acute lower extremity deep venous thrombosis diagnosed by duplex in patients hospitalized for coronavirus disease 2019. Journal of Vascular Surgery: Venous and Lymphatic Disorders.

77. Fox TA, Troy-Barnes E, Kirkwood AA, Chan WY, Day JW, Chavda SJ, et al. Clinical outcomes and risk factors for severe COVID-19 in patients with haematological disorders receiving chemo- or immunotherapy. British Journal of Haematology.n/a(n/a).

78. Mei F, Fan J, Yuan J, Liang Z, Wang K, Sun J, et al. Comparison of Venous Thromboembolism Risks Between COVID-19 Pneumonia and Community-Acquired Pneumonia Patients. Arteriosclerosis, Thrombosis, and Vascular Biology. 2020;40(9):2332-7.

79. Aleva FE, van Mourik L, Broeders MEAC, Paling AJ, de Jager CPC. COVID-19 in critically ill patients in North Brabant, the Netherlands: Patient characteristics and outcomes. Journal of Critical Care. 2020;60:111-5.

80. Fredi M, Cavazzana I, Moschetti L, Andreoli L, Franceschini F, Airò P, et al. COVID-19 in patients with rheumatic diseases in northern Italy: a single-centre observational and case&#x2013;control study. The Lancet Rheumatology. 2020;2(9):e549-e56.

81. Pizzolo F, Rigoni AM, De Marchi S, Friso S, Tinazzi E, Sartori G, et al. Deep vein thrombosis in SARS-CoV-2 pneumonia-affected patients within standard care units: Exploring a submerged portion of the iceberg. Thrombosis research. 2020;194:216-9.

82. Larsen K, Coolen-Allou N, Masse L, Angelino A, Allyn J, Bruneau L, et al. Detection of Pulmonary Embolism in Returning Travelers with Hypoxemic Pneumonia due to COVID-19 in Reunion Island. The American Journal of Tropical Medicine and Hygiene. 2020;103(2):844-6.

83. Chen S, Zhang D, Zheng T, Yu Y, Jiang J. DVT incidence and risk factors in critically ill patients with COVID-19. Journal of thrombosis and thrombolysis. 2020:1-7.

84. Ierardi AM, Coppola A, Fusco S, Stellato E, Aliberti S, Andrisani MC, et al. Early detection of deep vein thrombosis in patients with coronavirus disease 2019: who to screen and who not to with Doppler ultrasound? Journal of Ultrasound. 2020.

85. Soumagne T, Lascarrou J-B, Hraiech S, Horlait G, Higny J, d’Hondt A, et al. Factors Associated With Pulmonary Embolism Among Coronavirus Disease 2019 Acute Respiratory Distress Syndrome: A Multicenter Study Among 375 Patients. Critical Care Explorations. 2020;2(7):e0166.

86. Gatto M, Perricone C, Tonello M, Bistoni O, Cattelan AM, Bursi R, et al. Frequency and clinical correlates of antiphospholipid antibodies arising in patients with SARS-CoV-2 infection: findings from a multicentre study on 122 cases. Clin Exp Rheumatol. 2020;38(4):754-9.

87. Mazzaccaro D, Giacomazzi F, Giannetta M, Varriale A, Scaramuzzo R, Modafferi A, et al. Non-Overt Coagulopathy in Non-ICU Patients with Mild to Moderate COVID-19 Pneumonia. Journal of clinical medicine. 2020;9(6):1781.

88. Cattaneo M, Bertinato EM, Birocchi S, Brizio C, Malavolta D, Manzoni M, et al. Pulmonary Embolism or Pulmonary Thrombosis in COVID-19? Is the Recommendation to Use High-Dose Heparin for Thromboprophylaxis Justified? Thrombosis and haemostasis. 2020;120(8):1230-2.
